# Comparative Quantitative and Discriminant Analysis of Wheat Flour with Different Levels of Chemical Azodicarbonamide Using NIR Spectroscopy and Hyperspectral Imaging

**DOI:** 10.3390/foods13223667

**Published:** 2024-11-18

**Authors:** Hongju He, Yuling Wang, Shengqi Jiang, Jie Zhang, Jicai Bi, Hong Qiao, Leiqing Pan, Xingqi Ou

**Affiliations:** 1 School of Food Science, Henan Institute of Science and Technology, Xinxiang 453003, China; bijicai1983@163.com; 2 School of Life Science & Technology, Henan Institute of Science and Technology, Xinxiang 453003, China; wangyuling634@163.com; 3 College of Food Science and Engineering, Northwest A&F University, Yangling 712100, China; shq_jiang@163.com; 4 Henan Xinlianxin Chemical Industry Co., Ltd., Xinxiang 453003, China; jieguai1988@163.com; 5 Henan Shudiyi Seed Industry Co., Ltd., Xinxiang 453003, China; qiaohong163@163.com; 6 College of Food Science and Technology, Nanjing Agricultural University, Nanjing 210095, China; pan_leiqing@njau.edu.cn

**Keywords:** comparative analysis, wheat flour, azodicarbonamide, NIR, hyperspectral imaging

## Abstract

This study investigated and comprehensively compared the performance of spectra (950–1660 nm) acquired respectively from NIR and HSI in the rapid and non-destructive quantification of azodicarbonamide (ADA) content (0–100 mg/kg) in WF and simultaneously identified WF containing excessive ADA (>45 mg/kg). The raw spectra were preprocessed using 14 methods and then mined by the partial least squares (PLS) algorithm to fit ADA levels using different numbers of WF samples for training and validation in five datasets (N_Training_/_Validation_ = 189/21, 168/42, 147/63, 126/84, 105/105), yielding better abilities of NIR Savitzky–Golay 1st derivative (SG1D) spectra-based PLS models and raw HSI spectra-based PLS models in quantifying ADA with higher determination coefficients and lower root-mean-square errors in validation (R^2^_V_ & RMSEV), as well as establishing 100% accuracy in PLS discriminant analysis (PLS-DA) models for identifying excessive ADA-contained WF in each dataset. Twenty-four wavelengths selected from a NIR SG1D spectra in a 168/42 dataset and 23 from a raw HSI spectra in a 147/63 dataset allowed for the better performance of quantitative models in ADA determination with higher R^2^_V_ and RMSE_V_ in validation (R^2^_V_ > 0.98, RMSE_V_ < 3.87 mg/kg) and for discriminant models in WF classification with 100% accuracy. In summary, NIR technology may be sufficient if visualization is not required.

## 1. Introduction

Wheat, as one of the most important and indispensable cereal crops, is widely cultivated all over the world, with its production areas mostly concentrated in Asia, Europe, and the Americas, occupying 43.7%, 32.9%, and 16.7% of total world yields in the years 1994–2021, respectively, according to the latest statistics of Food and Agriculture Organization of the United Nations [1]. Harvested wheat is mainly ground into flour [2] and then used as a good raw material to produce various foods, such as bread, noodles, snacks, cakes, pastries, and other flour-based products [3]. Wheat flour (WF) contains multiple ingredients such as protein, fat, carbohydrates, fiber, and various vitamins and minerals, providing essential nutrients for human growth and development [4]. To meet the requirements for producing different WF-based foods, some additives acted as gluten reinforcing agents, improving agents, whitening agents, gluten-reducing agents, and starter cultures are often used to improve WF characteristics and functions in industrial processing of WF [5].

Among the permitted food additives, azodicarbonamide (ADA), a synthetic chemical compound characterized by yellow, odorless, and low-cost properties, is widely used and mainly plays a role in conditioning dough or bleaching flour in WF-based food production [6]. As a dough conditioner, ADA can improve the properties of dough, making it more elastic and easier to use in processing of bread and pastry. Furthermore, ADA also can strengthen the dough by improving its texture, resulting in bread and baked foods with better taste [7]. As a flour bleaching agent, ADA helps to whiten WF by reducing the yellow color that often appears during storage and is especially important for some WF-based products such as white bread and some pastries that are visually expected to carry bright white appearances [8]. In many countries, including the United States, China, Canada, Brazil, and Korea, ADA is generally accepted and recognized as a safe food additive when its usage is within a reasonable limit, causing no harm to human health [9]. According to regulations in these countries, the maximum allowed usage of ADA in WF is 45 mg/kg. However, some other regions or countries such as the European Union, Australia, and Singapore have banned the use of ADA because of some research results indicating that ADA can be broken down into semicarbazide and urethane during the baking process, and the two decomposition compounds may cause cancer and thus raise health concerns [10], although ADA is currently listed as a legal food additive by the United Nations Codex Alimentarius Commission and the US Food and Drug Administration. In practical WF production, some enterprises or manufacturers, driven by great commercial interests, add excess ADA to WF, which is illegal and unacceptable. The ADA concentration in WF should be strictly monitored by regulatory authorities to ensure that the WF is safe to be consumed and that consumer rights and interests are not infringed. Therefore, it is very necessary to provide technical support for the rapid detection and identification of ADA content in WF by developing and applying advanced technologies.

Common techniques to quantify the amount of ADA in foods mainly includes high-performance liquid chromatography (HPLC) [6], electrophoresis [11], gas chromatography-mass spectrometry (GC-MS) [12], colorimetric methods [13], fluorescent probe [14], enzyme-linked immunosorbent assay (ELISA) [15], and nuclear magnetic resonance (NMR) spectroscopy [16], which are destructive, tedious, time-consuming, reagent-based, environmentally unfriendly, and also require well-trained personnels to complete the whole procedure. Optical methods such as near-infrared (NIR) spectroscopy and hyperspectral imaging (HSI) have been proved to be great potential and powerful in food quality evaluation [17,18], and have obvious characteristics of rapid, non-invasive, green, reagent-free, pollution-free, and easy to operate. Both NIR and HSI are spectra-based analytical techniques and can provide spectral information related to a target index to perform quantitative and qualitative analysis. Different from NIR technology, HSI combines spectroscopy and imaging simultaneously, and is capable of offering detailed spectral information at each pixel of a two-dimensional image [19], which means that more information on a target parameter can be obtained from HSI. As NIR and HSI equipment originated from different manufacturers and show different performance levels, the differences between NIR and HSI in determining ADA levels in WF and identifying whether WF contains excessive ADA levels have never been comparatively studied and analyzed [20]. Through comparative analysis, the accuracy, sensitivity, and stability of NIR and HSI in the determination of ADA in WF can be evaluated, and a more suitable one can be selected to improve the detection efficiency, which is quite conducive to making a right decision to adopt NIR or HSI in practical applications. Furthermore, it can provide scientific basis and technical support for food safety supervision departments to help monitor ADA content in WF more effectively and ensure the implementation of food safety regulations, by comparing and analyzing the performance of the two technologies.

**Figure 1:**
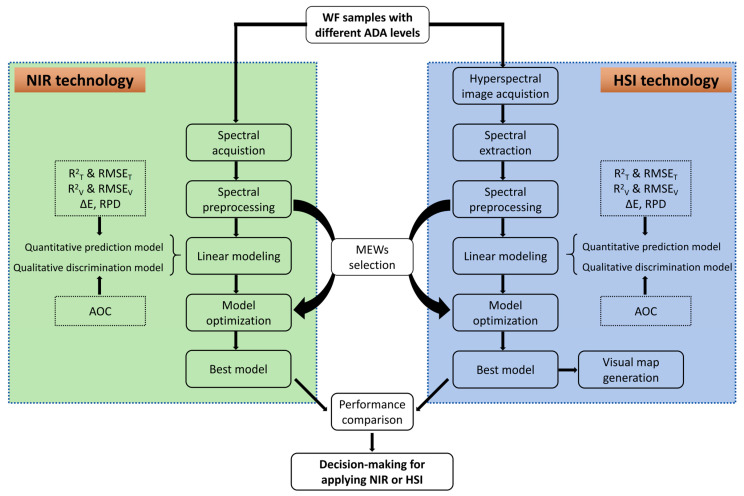
The flow chart of NIR and HSI for quantitative and discriminant analysis of wheat flour with different levels of ADA.

In view of the lack of current comparative studies, to have a deeper and more comprehensive understanding of the detection performance differences between NIR spectroscopy and HSI, we proposed to investigate and compare the abilities of the two technologies within the same spectral range in analyzing ADA levels in WF quantitatively and qualitatively, providing technical and decision-making support to determine whether NIR or HSI is more appropriate. The same range of wavelength information was obtained from the two technologies and their capabilities in ADA prediction by linear modeling and supervised discriminant analysis of WF containing different levels of ADA were compared. In this study, the specific research objectives were as follows: (1) acquire the spectral information of WF samples (WF with different ADA levels) from NIR and HSI in the same spectral range, (2) extract and preprocess the acquired spectra using different methods, and pick out the most appropriate preprocessing method, (3) build quantitative relationships between the spectra and the ADA levels using linear algorithms, and evaluate the models performance, (4) build discriminant analysis (DA) models to identify WF with permitted ADA levels or with excess ADA levels, (5) select the most effective wavelengths (MEWs) to simplify the original model, and compare the simplified models performance in ADA quantization and WF classification, (6) generate color maps to visually observe the differences of WF containing different ADA contents by transferring a best simplified model, (7) comprehensively evaluate, compare, and summarize the differences between NIR and HSI in the quantitative and qualitative analysis of ADA levels in WF. The flow chart for investigating the performance difference of the two technologies is shown in Figure 1.

## 2. Materials and Methods

### 2.1. WF Samples Preparation

Wheat seeds (variety, Bainong 607) were ground into flour (moisture, 12 ± 0.5%) using an experimental mill (CD1, Chopin, France), that is WF (same as commercial flour, 80 mesh), and then stored in a stable environment (humidity, 60%). The ADA powder (Sigma-Aldrich, St. Louis, MO, USA) was added in the WF to prepare WF samples with different concentrations of ADA with the help of a balance holding an accuracy of 1/10,000. A flip mixer (HF-12, HerryTech, Shanghai, China) was used to mix the WF and the ADA thoroughly. To meet the modeling requirements using a wide range of ADA values, the ADA concentration gradients in WF were set as 0–100 mg/kg (intervals, 5 mg/kg), resulting in 21 different ADA levels. Ten duplicate WF samples for each ADA level were prepared and a total of 210 WF samples were finally obtained.

### 2.2. Spectral Acquisition from NIR and HSI Equipments

In this study, a NIR device (Version, NIRez education-kit, Isuzu Optics Corp., Taiwan) and a HSI system (Version, HSI-eNIR-XC130, Isuzu Optics Corp., Taiwan) were used. The NIR device consists of a ring-shaped halogen illuminator (20 W), a spectrograph (InGaAs detector, 1 mm) covering a wavelength range of 901.76–1700.66 nm (intervals, 1.30–2.61; 400 wavelengths), and software (Version, Isuzu Optics NIRez 2.0 Rice). The exposure time was set as 0.63 ms, and the number of scans to average was set as 5 when using the NIR device. The HSI system mainly contains five components in a black box, such as a CCD camera (Version, DL-604 M, Andor, Ireland) carrying a zoom lens (Version, OLE2, Schneider, Germany), a spectrograph (Version, ImSpector V10E, Spectral Imaging Ltd., Oulu, Finland) offering spectral range from 879.132 nm to 172.510 nm with waveband intervals of 1.7 nm (900–1700 nm is suggested by manufacturer), two halogen illuminators (Illumination Technologies Inc., Liverpool, NY, USA), an electric-controlled mobile platform (Version, IRCP0076-1COMB, Isuzu Optics Corp, Taiwan), and an aluminum alloy support frame holding and connecting the four parts mentioned above. Outside the box, a high-speed computer is essential and installed with two special-purpose software applications, namely Spectral Image and Analyzer (Isuzu Optics Corp, Taiwan). Two parameters, including exposure time and platform moving speed, in the HSI system were set as 3.80 ms and 7.45 mm/s, respectively. The two different types of equipment were preheated for 30 min before each test to ensure their stable operation in the whole process of spectral acquisition.

The NIR instrument was calibrated via scanning a white tile bar (~99.9% reflectance) and turning off the illuminator (0% reflectance), which was completed using the Optics NIRez software (Version, 2.0 Rice). As for the HSI system, a white image (I_W_) and a dark image (I_D_) were obtained first through scanning a white tile bar (~99.99% reflectance) and covering the lens completely with its cap (0.00% reflectance), respectively, to perform calibration. Each WF sample was then put into a glass dish (diameter, 60 mm; height, 10 mm) and scanned successively by the NIR device and the HSI system. The raw hyperspectral images (I_R_) of WF samples acquired from the HSI system were calibrated into images (I_C_) reflecting reflectance using the following Equation (1), which was performed in the Spectral Image software (Version, 2.0 Rice).

(1) $$I_{C}=\frac{I_{R}-I_{D}}{I_{W}-I_{D}}\times 100\%$$

**Figure 2:**
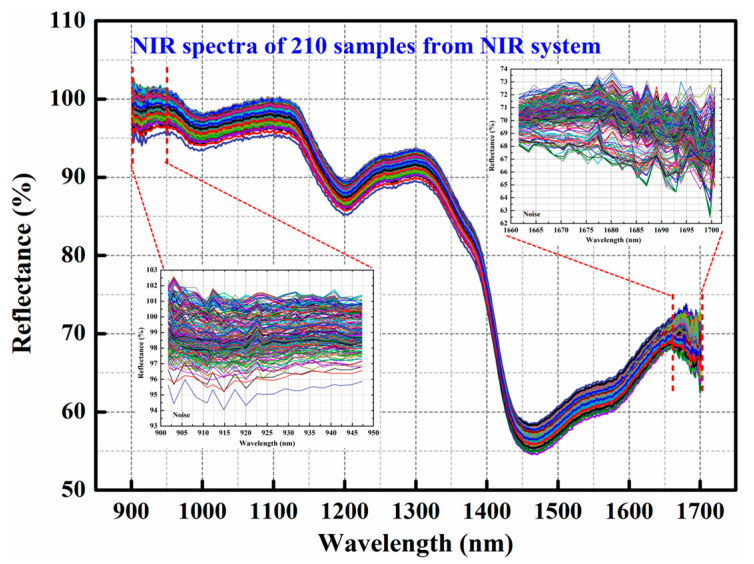
The full raw spectral features of all WF samples acquired from NIR device.

The spectral information in every pixel within the region of interest (ROI) of each calibrated hyperspectral image was extracted and averaged as one spectrum, which was carried out using Analyzer software (Version, 2.0 Rice). The spectra obtained from the two different instruments were separately arranged in Origin 8.5 software (OriginLab Corporation, Northampton, MA, USA). The obvious noise was found in two ranges of 901.76–947.27 nm and 1661.88–1700.66 nm from the NIR device, which is shown in Figure 2. In order to ensure the rationality and comparability of the results of spectral data analysis, the spectral information in the same range of 950–1660 nm from the two instruments was used for further processing.

### 2.3. Spectral Preprocessing

Acquired raw NIR spectra often contain noise, baseline variations, and other artifacts that can affect the reliability of analytical results. These adverse effects are generally required to be minimized or even eliminated after data preprocessing. The spectral preprocessing has been a crucial step in spectral analysis, aiming at improving the quality of spectral data and facilitating subsequent data analysis and interpretation [21]. In this study, the 14 common techniques including absorbance (ABS), Kubelka–Munk (KM), normalization (NOR), multiple scattering correction (MSC), baseline correction (BC), standard normal variables (SNV), moving average smoothing (MAS), Savitzky–Golay smoothing (SGS), median filter smoothing (MFS), Gaussian filter smoothing (GFS), mean center (MC), Savitzky–Golay-1st-derivative (SG1D), Savitzky–Golay-2nd-derivative (SG2D), and de-trending (DT) [22], were applied to preprocess the raw 950–1660 nm range spectra of WF samples, which was executed using Unscrambler X software (Version 10.4, CAMO, Oslo, Norway).

### 2.4. Quantitative Model and Discriminant Analysis Model Construction and Evaluation

After spectral preprocessing, the raw and preprocessed spectra from the NIR device and the HSI system were respectively mined by partial least squares (PLS) algorithm to fit the ADA levels, constructing mathematical models to achieve the quantitative prediction of ADA level in WF samples. PLS is a multivariate statistical technique commonly used for regression and dimensionality reduction in data analysis, and particularly suitable in fields like spectroscopy. PLS aims to find a linear relationship between a set of independent X-variables (e.g., NIR spectra) and a set of dependent Y-variables (e.g., ADA concentrations) by creating a set of new variables, known as latent variables (LVs) or components that capture the most relevant information in the data [23]. PLS discriminant analysis (PLS-DA), as an extension of PLS regression, is mainly used for supervised classification and discrimination purposes. PLS-DA is widely applied in fields such as chemometrics, bioinformatics, and pattern recognition for tasks such as quality control, disease diagnosis, and classifying samples into different categories based on multivariate data [24].

In this study, by inputting the NIR spectra and the ADA values into a matrix, and then executing the PLS modeling program and the PLS-DA modeling program in the Unscrambler X software, the PLS models for quantifying ADA levels in WF samples and the PLS-DA models for identifying WF samples carrying excess ADA concentrations were respectively established. The performance of the PLS model was evaluated in terms of determination coefficients (R^2^) and root-mean-square errors (RMSE) in the training set (R^2^_T_ & RMSE_T_) and validation set (R^2^_V_ & RMSE_V_). The absolute value between RMSE_T_ and RMSE_V_ (ΔE), the prediction bias, and the residual predictive deviation (RPD) were also calculated and used to assist in the evaluation of PLS model capability. The accuracy of classification (AOC) was applied to assess the DA effect with PLS-DA models. Generally, a PLS model showing a good performance should have higher values of R^2^_T_, R^2^_V_, and RPD, and lower values of RMSE_T_, RMSE_V_, ΔE, and prediction bias. In addition to the higher R^2^ and lower RMSE, a larger AOC value also indicates the good ability of the PLS-DA model [25].

### 2.5. MEWs Selection and Model Simplification

NIR spectra often contain a wide range of wavelengths and sometimes carry hundreds of wavelengths, i.e., high dimensionality, which always results in an increase in data computation load and overfitting in predictive models [26]. Furthermore, not all wavelengths in an NIR spectrum contribute equally to the information regarding the chemical composition or properties of a sample. Only parts of wavelengths are related to the prediction of sample properties [27]. Therefore, selecting a set of MEWs holding small numbers of wavelengths is necessary and can help to remove uninformative or redundant spectral variables. A small number of MEWs is easier to interpret and relate to a specific target chemical or physical property of a sample, which is valuable for understanding the prediction mechanism and reducing the costs and efforts required for data acquisition. In addition, spectral dimensionality reduction through MEW selection can also make spectral analysis faster and more manageable. On the other hand, a model built with MEWs may perform better in terms of prediction accuracy, generalization, and model interpretability, as well as tend to be simpler and faster to train and apply, which is particularly advantageous in real-time or process monitoring applications where a quick decision is required [28].

The MEWs in the present study were selected by the combination of stepwise regression and PLS β-coefficients (SRC). Stepwise regression is a statistical method used for selecting a subset of independent X-variables (i.e., NIR wavelengths) from a larger set of X-variables in a multiple regression model, identifying the most relevant X-variables that significantly contribute to explaining the variation of dependent Y-variable (i.e., ADA levels) and removing the less relevant ones at the same time. Stepwise regression for selecting MEWs involves two main procedures, forward selection and backward elimination [29], which were executed in the Matlab software (Version R2016a, The MathWorks, Inc., Natick, MA, USA). Similarly, β-coefficients, also known as regression coefficients or loadings, represent the relationships between independent X-variables and dependent Y-variable, indicating the contribution of each X-variable to the prediction of Y-variable. The wavelengths corresponding to large absolute coefficient values (regardless of signs) are often considered to make great contributions to the prediction and should be selected as MEWs [30], and this was performed using the Unscrambler X software (Version 10.4).

Next, the selected MEWs were used for input as new X-variables to build simplified PLS models to predict ADA levels in WF samples and simplified PLS-DA models to classify WF samples with or without ADA levels in excess, respectively. In addition to PLS regression, multiple linear regression (MLR) is also applicable when the number of X-variables is smaller than that of Y-variables [31]. The simplified PLS and MLR models, and AD models including PLS-DA and MLR-DA models, were evaluated using the same parameters mentioned above and compared to select a best one for the subsequent external test and spatial visualization.

### 2.6. Independent External Validation

External validation using an independent set of samples (test set) is a crucial step in further assessing a model’s performance, reliability, and generalization to new unknown data. External validation ensures that the established model can achieve accurate predictions on new independent data, which is crucial for the practical applicability of the model. Moreover, external validation helps to prevent the model from being over-adapted to the training data and provides a more realistic and objective assessment of the model’s real-world performance [32]. In this study, a set of independent WF samples with different ADA levels were collected to validate the selected best simplified PLS model and PLS-DA model.

### 2.7. Spatial Visualization of ADA Concentrations

Visualization is an obvious advantage function of the HSI technology and assists in understanding and interpreting the spectral information captured by hyperspectral sensors. After the model simplification, the selected best linear model was transferred into each pixel of the original calibrated ROI images of each sample to form color distribution maps by calculating a dot product between the regression coefficients of the model and the spectrum of each pixel within the ROI image, which was completed in Matlab software. In the process, the pixels showing similar spectral profiles at the MEWs generated similar color magnitudes in the ROI image. With the color maps, the WF samples with different ADA levels were visually observed and the ADA distribution in WF samples were well interpreted via spectral information conversion.

## 3. Results

### 3.1. Raw and Preprocessed Spectral Features of WF Samples

**Figure 3:**
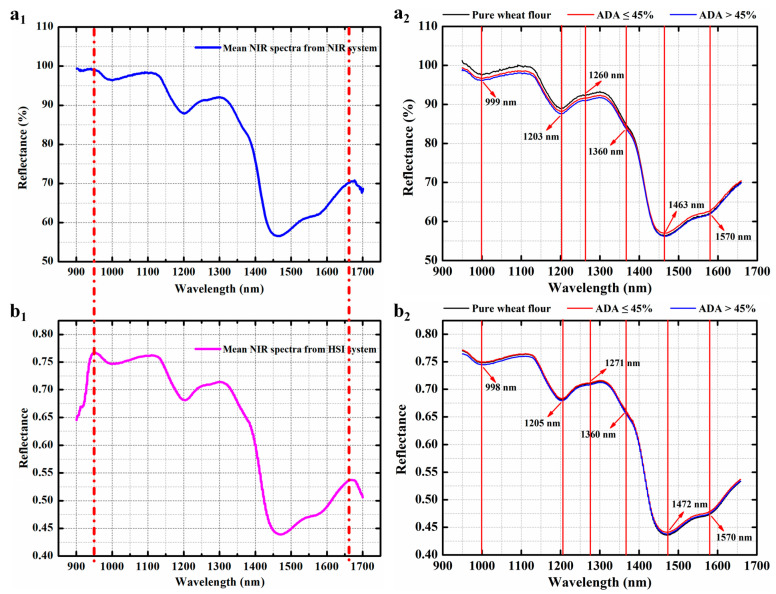
The full mean spectral features of all WF samples acquired from NIR device (**a_1_**,**a_2_**) and HSI system (**b_1_**,**b_2_**), respectively.

The raw mean spectral characteristic curves of all WF samples (Figure 3(a_1_,b_1_)), pure WF samples, WF samples with ADA levels ≦45 mg/kg, and WF samples with ADA levels >45 mg/kg (Figure 3(a_2_,b_2_)) in the full region of 950–1660 nm are given and shown in Figure 2, and the preprocessed ones in the same spectral range are exhibited in Figure S1.

As can be seen, the variation tendencies of the preprocessed spectral profiles of WF samples were almost the same, regardless of applying any of the 14 preprocessing methods. At the same time, it was noticed that the heights of all spectral curves were different in raw and preprocessed spectra, which was probably due to the different ADA concentrations in WF samples. Through detailed observation and analysis, it was found that the several absorption peaks of WF samples appeared with strong absorption at ~999 nm (O–H stretching overtones and combination), ~1203 nm (second overtone of C–H stretching vibration), and ~1463 nm (first overtone of O–H stretching vibration) [33], as well as weak absorptions at ~1260 nm (second overtone of C–H stretching vibration) [34], ~1360 nm (combination of first and second overtones of C–H stretching vibration) [35], and ~1570 nm (first overtone of N–H stretching vibration) [36], after absorbing NIR light emitted from the NIR device. Similar absorption peaks of WF samples were observed after absorbing the emitted NIR light from the HSI system.

Nevertheless, no obvious characteristic peak was found to represent the ADA absorption in the range of 950–1660 nm. By using appropriate chemometric means, the latent relationship between the NIR spectra and the ADA values could be established to achieve the rapid quantification of ADA in WF. In other words, the spectral information related to ADA prediction in the 950–1660 nm range could be mined by proper chemometric methods and used for quantifying ADA.

### 3.2. Quantifying ADA and Identifying WF Samples Using Spectra Originated from NIR

A certain number of WF samples were randomly selected from each ADA concentration gradient and used as a validation set (V), and the remaining WF samples were classified into a training set (T), leading to five groups of N_T/V_ datasets (N_T/V_ = 189/21, N_T/V_ = 168/42, N_T/V_ = 147/63, N_T/V_ = 126/84, N_T/V_ = 105/105) for modeling and comprehensively evaluating the performance of established PLS models in quantifying ADA concentrations in WF samples and PLS-DA models in discriminating WF samples with or without excess ADA levels. The detailed results by applying spectra from the NIR device are shown in Table S1, and the best ones are displayed in Table 1.

By analyzing and comparing the results of the PLS models built with the full 5 raw and 70 preprocessed spectra of 950–1660 nm originated from the NIR device in the five N_T/V_ groups, it was found that the 75 PLS models had really good performances in predicting ADA contents in the WF samples (R^2^_V_ = 0.9439–0.9943, RMSE_V_ = 2.28–7.17 mg/kg) (Table S1). By comparison, for each N_T/V_ group, the PLS model based on SG1D spectra performed better in predicting ADA content with a smaller number of LVs (6) involved to generate relatively higher values of R^2^ and RPD, as well as lower values of RMSEs and ΔE (highlighted in Table 1), than the PLS models based on raw spectra and other preprocessed spectra in each N_T/V_ group. The results indicated that preprocessing of the spectra from the NIR device was indeed necessary and had a certain impact on the PLS model performance with different preprocessing methods leading to different results of predicting ADA in WF samples using the same N_T/V_ group dataset. The SG1D method was considered more suitable for preprocessing the 950–1660 nm range spectra than the NIR device, as it improved the predictive ability of the raw spectra-based PLS model in detecting ADA levels in the WF samples to the greatest extent. In addition, it was also observed that there were small differences among the same spectra-based PLS models in the five N_T/V_ groups, revealing that the number of WF samples used for training and validation had some effects on model performance.

Based on the same raw and preprocessed spectra, PLS-DA models were also established to classify the WF samples into two categories: WF with permitted ADA and WF with excess ADA, achieving 100% AOC in validation set for all the 15 models in each N_T/V_ group. The spectral preprocessing and the WF numbers in the training set and validation set did not affect the AOC of the supervised PLS-DA models.

So far, a few studies on the use of NIR technology to detect ADA levels in WF have been reported by other researchers. Gao et al. [37] studied an 850–1050 nm spectra of WF using a non-linear radial basis function (RFB) algorithm to achieve ADA detection with excellent accuracy (R = 0.9949, RMSE = 2.0286 mg/kg). The same algorithm also gave a similarly good performance in detecting ADA (R = 0.99996, RMSE = 0.5467 mg/kg) in a 400–2500 nm range [38]. However, WF samples for internal prediction and external testing were not provided in these reported works, which weakened the accuracy and reliability of the RFB model in the evaluation of ADA content in WF. Although Du et al. [39] used different numbers of WF samples for model calibration and prediction in ADA detection, giving an R^2^ of 0.99814 and an RMSE_P_ of 2.91345 mg/kg, a set of independent WF samples were not applied to validate the predictive model externally, which is quite important to the applicability and validity of a calibration model. Compared to these studies, although we achieved slightly weaker results that may be due to the different spectral range, preprocessing methods, and different numbers of WF samples and ADA concentrations involved in this study, different preprocessing methods were used and their effects on model accuracy were investigated and analyzed comprehensively, which provided more information to understand the differences of various spectral preprocessing techniques in improving model performance. In addition, a linear algorithm was used to mine spectra, and that was different from the three above-mentioned studies using nonlinear algorithms, which indicated that there were both linear and nonlinear relationships between NIR spectra and ADA concentration values in WF. In addition, different numbers of WF samples were used in model training and validation, and the impacts of different WF sample sizes on predicting ADA levels were compared and analyzed. More significantly, we established supervised DA models to identify whether WF contained ADA in excess and achieved 100% accuracy, which was not investigated in the three published studies. In other words, the use of NIR technology to analyze WF containing different ADA contents can be extended for qualitative analysis in addition to quantitative detection.

In summary, the present research results were more comprehensive and offered more abundant information for interpreting the NIR spectra of WF at different ADA levels, which can lead to a deeper understanding of the use of NIR technology for WF analysis. Moreover, these findings provided valuable data and technical support for future industrial applications, whether in terms of ADA quantification alone or identification of excess ADA-contained WF alone, or both the quantitative and qualitative analysis at the same time.

### 3.3. Quantifying ADA and Identifying WF Samples Using Spectra Originated from HSI

As shown in Table 1, by applying the spectra from the HSI system, 75 PLS models were built using the five N_T/V_ groups of datasets to produce similarly good predictive results with an R^2^_V_ of 0.9459–0.9876 and an RMSE_V_ of 3.3704–7.0390 mg/kg, which were comparable to the PLS models using spectra from the NIR device. By comparison, the raw spectra-based PLS model in each N_T/V_ group had a slightly better ability in determining ADA content than other spectra-based PLS models (highlighted in Table 1). The results indicated that the applications of the 14 preprocessing methods did not improve the PLS model’s performance in the quantitative prediction of ADA content in WF samples, which was probably due to the spectral average operation for all pixels of hyperspectral images itself playing a role of preprocessing in the process of spectral extraction. This means that in the present study, it was not necessary to preprocess the spectra obtained by the HSI system, as the results were unsatisfactory. With the same raw and 14 preprocessed spectra, 15 PLS-DA models were also established to classify the WF samples, yielding 100% AOC in the validation set for each N_T/V_ group, except for the KM spectra-based PLS-DA model in the N_T/V_ = 147/63 group, and the SD1D and SG2D spectra-based PLS-DA models in the N_T/V_ = 126/84 group. In a word, the raw spectra were more suitable for detecting ADA levels and identifying WF with ADA in excess.

In addition, a small performance difference was found between the two PLS models built with spectra (from NIR and HSI) preprocessed by same technique in same N_T/V_ group, which indicated that the spectra acquired from the NIR device and HSI system had an effect on PLS models ability in detecting ADA and that may be caused by the different numbers of wavelengths and different individual wavelength in the two different pieces of equipment.

At present, there are very few reports on the use of hyperspectral imaging to detect ADA content in WF. By searching the latest literature, it was found that only Wang et al. [40] applied HSI to detect ADA in WF. Through analyzing the hyperspectral images of pure WF, pure ADA, and WF-ADA mixtures (ADA content, 0.2–10 g/kg), using a band ratio algorithm combined with threshold segmentation, a linear relationship between ADA content and the number of ADA-rich pixels was established to produce a correlation coefficient of 0.9845, providing a methodological support for the detection of ADA in WF. By comparison, in this study, we built models to analyze ADA-contained WF based on spectra rather than image information. It also indicated that both spectral information and image information provided by HSI could be correlated with ADA contents. More in-depth and extensive studies on the application of HSI to control WF quality are still required.

To facilitate the rapid quantitation and discriminant analysis of WF samples holding different ADA levels simultaneously, it is highly recommended to apply the same spectra in modeling for quantitative and qualitative analysis. In the present study, the SG1D spectra and the raw spectra acquired from the NIR device and HSI system, respectively, were selected for further wavelength selection and model simplification.

### 3.4. MEWs Selected by SRC Method

To accelerate the prediction of ADA levels in WF samples and the identification of ADA-contained WF samples, a smaller number of wavelengths carrying the most effective information, that is, MEWs, were expected and selected by the SRC method. The specific MEWs selected from SG1D spectra (originated from NIR) and from raw spectra (originated from HSI) are shown in Table S2, and the most appropriate MEWs are exhibited in Table 2.

For the SG1D spectra, the numbers of MEWs selected from the five N_T/V_ groups were different, with a total of 22–30 MEWs retained after wavelength selection. In other words, the 355 wavelengths in the full 950–1660 nm range reduced by 92–94%, with less than 10% of the wavelengths staying after screening MEWs. Similarly, for the raw spectra from the HSI system in the five N_T/V_ groups, a number of 23–30 different MEWs were selected, and that accounted for only 5–7% of the total 432 wavelengths. For the same N_T/V_ group, it was found that the numbers of the MEWs selected from the SG1D spectra (originated from NIR) and the raw spectra (originated from HSI) were different (N_T/V_ = 126/84 group excluded) and varied by 2–3. The most specific individual wavelengths in the same N_T/V_ group were also different.

The results indicated that a change in the number of WF samples in the training set and validation set led to a change in the number of MEWs and the specific wavelengths for either the SG1D spectra from NIR or the raw spectra from HSI. In other words, the number of WF samples used for model training and validation had an impact on the MEWs in terms of number and individual wavelength.

### 3.5. Quantifying ADA Levels and Identifying WF Samples Using MEWs

By inputting the MEWs as new X-variables and the same ADA values as Y-variables to execute PLS algorithm and MLR algorithm, respectively, the five initial PLS models based on full-range SG1D spectra were simplified, and the MLR models were also constructed. The DA models based on the MEWs were also established. Their individual performances in analyzing WF samples with different ADA contents are shown in Table S3, and the best results are exhibited in Table 3.

Based on the MEWs selected from the SG1D spectra for each N_T/V_ group, although the simplified PLS model had a similar good performance compared with the corresponding full-range SG1D spectra-based PLS model using the same N_T/V_ group dataset, the established MLR model performed better in predicting ADA content with a higher R^2^_V_ and RPD, as well as a lower RMSE_V_ and prediction bias. Additionally, the MLR model based on 24 MEWs in the N_T/V_ = 168/42 group possessed a better capability than other simplified PLS models and MLR models, with a R^2^_V_ of 0.9898, a RMSE_V_ of 3.0633 mg/kg, and a RPD of 9.9001. Based on the same MEWs in each N_T/V_ group, the MLR-DA model still yielded 100% AOC in WF classification while the simplified PLS-DA model achieved less than 100% AOC. The results indicated that the use of MEWs did not reduce the predictive accuracy of PLS models but decreased the AOC of PLS-DA models. In comparison, the MEWs enabled the MLR model to perform better in ADA prediction and kept the MLR-DA model with the same 100% AOC in WF identification for the same N_T/V_ group dataset.

Similar results were also obtained after applying the MEWs selected from the raw spectra (originated from HSI) to simplify the models. Still, based on the same number of MEWs, the MLR model performed slightly better than the PLS model, and the MLR-DA model had a slightly better performance than the PLS-DA model. The 23 MEWs from the N_T/V_ = 147/63 group dataset generated a better performance in detecting ADA by MLR model (R^2^_V_ = 0.9837, RMSE_V_ = 3.8633 mg/kg, RPD = 7.8388) and a 100% AOC in identifying WF by MLR-DA model.

In summary, based on the same numbers of MEWs, the MLR algorithm performed better and was more suitable for modeling to quantify the ADA level in WF and identify WF with different ADA levels (whether the ADA content in WF exceeded 45 mg/kg). The MLR model and the MLR-DA model using the 24 MEWs from NIR (defined as SRC-SG1D-MLR and SRC-SG1D-MLR-DA) were slightly more effective than using the 23 MEWs from HSI (defined as SRC-RAW-MLR and SRC-RAW-MLR-DA) and used for further analysis.

### 3.6. Testing Model Using External Independent Samples

**Figure 4:**
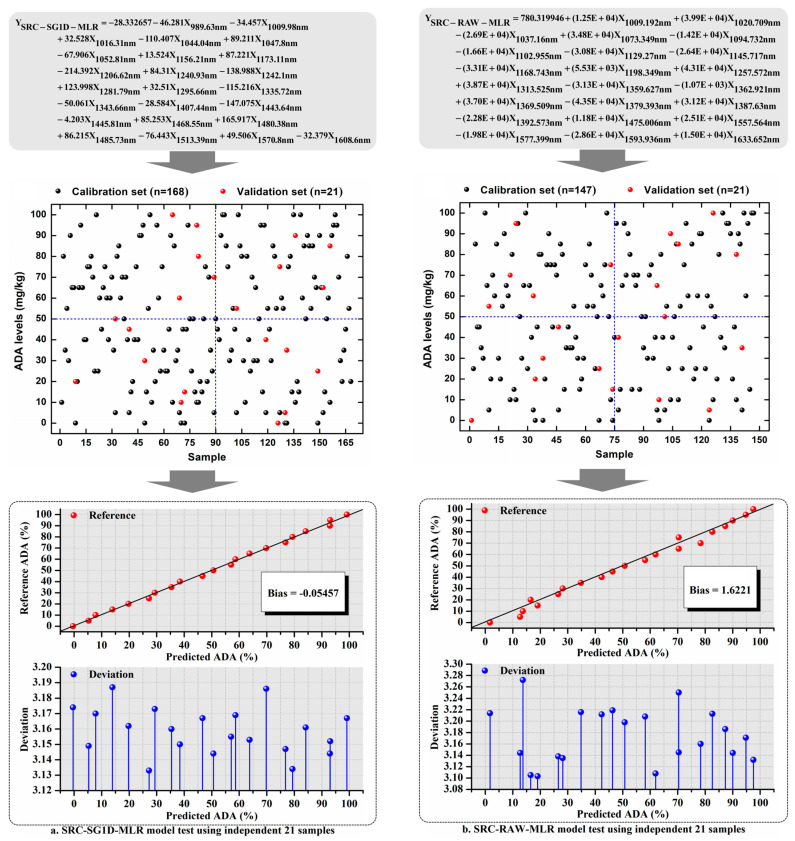

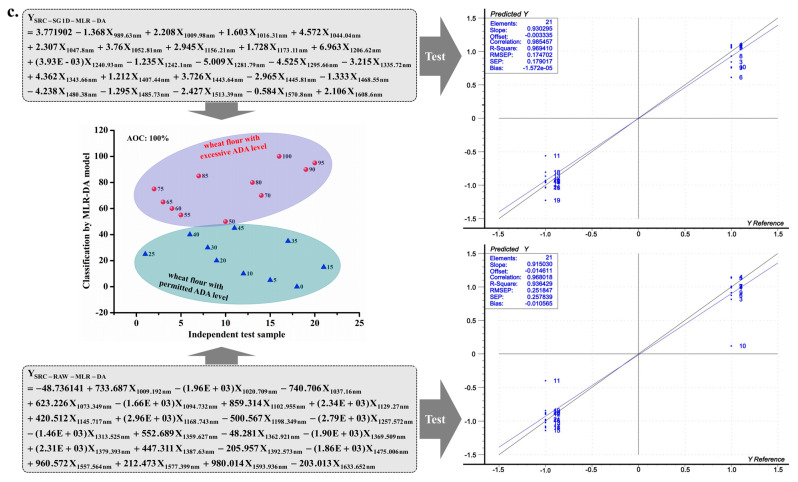
The external validation performance of SRC-SG1D-MLR model (**a**) and SRC-RAW-MLR model (**b**) for prediction, as well as the corresponding DA models (**c**) for classification using independent samples.

A set of independent WF samples (*n* = 21) with different ADA contents were prepared to test the model performance, and the final results on ADA quantification and WF classification are shown in Figure 4. The two MLR models for quantitative purposes were expressed as Y_SRC-SG1D-MLR_ (Figure 4a) and Y_SRC-RAW-MLR_ (Figure 4b). As seen from Figure 4a,b, the ADA concentrations in the 21 WF samples were predicted by the SRC-SG1D-MLR model with a deviation of 3.133–3.187 mg/kg (Figure 4a), and by the SRC-RAW-MLR model with a deviation of 3.103–3.272 mg/kg (Figure 4b), respectively. In addition, a higher R^2^_T_ and a lower prediction bias were found in the SRC-SG1D-MLR model, which indicated that the SRC-SG1D-MLR model performed better than the SRC-RAW-MLR model in quantifying ADA content in the WF samples.

The corresponding two MLR-DA models for discriminant purpose were expressed as Y_SRC-SG1D-MLR-DA_ and Y_SRC-RAW-MLR-DA_, which are shown in Figure 4c. It can be seen that the same 21 WF samples were correctly classified into two different categories, WF with ADA levels ≦45 mg/kg and WF with ADA levels >45 mg/kg, by the SRC-SG1D-MLR-DA model and the SRC-RAW-MLR-DA model, and 100% AOC was achieved for both the two DA models.

In short, the two quantitative models and the two DA models were all well validated externally, which meant that all the established models were robust and could be used in future industry applications. In other words, both the NIR and HSI had great potential in detecting ADA levels in WF.

### 3.7. Visualization of ADA Concentrations in WF Samples

**Figure 5:**
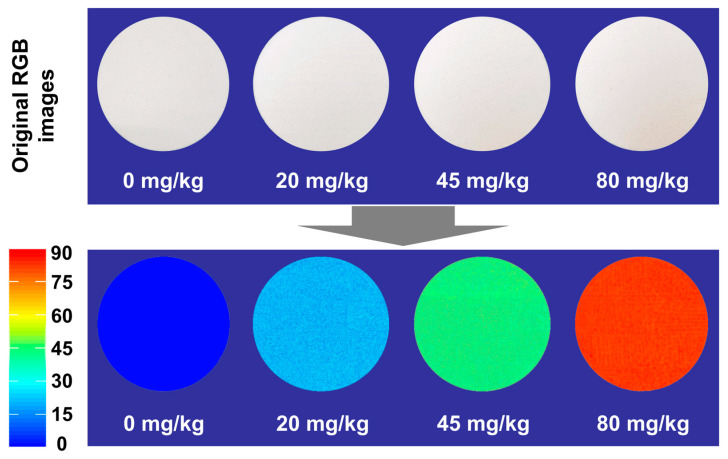
The example distribution maps of WF samples with different ADA levels.

The HSI technique has an advantage over the NIR technique due to the imaging function. In this study, the SRC-RAW-MLR model was transferred into every pixel of the original ROIs within the calibrated HSI images of WF samples with the help of a developed imaging algorithm, and the color maps displaying the distribution of different ADA concentration gradients among WF samples and the WF samples with different ADA levels were produced. Some examples are provided and illustrated in Figure 5.

As shown in Figure 5, the color scale on the left side with values ranging from small to large indicate that the color varied gradually from blue to red. The ADA content in each pixel in the maps and the different ADA contents in different WF samples from low to high were directly observed. Through the combination of the spectra and images in HSI, the ADA contents in the WF samples were quantitatively well predicted, and the WF samples with and without excess ADA levels were well visualized at the same time, which make the HSI more comprehensive in analyzing the ADA levels in WF samples, although the HSI performed slightly weaker than the NIR technology in the quantitative prediction of ADA.

NIR technology and HSI are both powerful tools to provide valuable insights into food quality, safety, and production processes. In industrial applications, the choice of which technology to use depends on the specific requirements and constraints of the food application. In fact, NIR technology is favored for its speed, cost-effectiveness, and ability to provide specific measurements crucial for food quality control and process optimization and is often used for rapid food analysis and composition determination in industrial settings [40]. HSI, while powerful for detailed spectral analysis, may be overkill for many routine food industry tasks where NIR spectroscopy can provide sufficient information for decision making. HSI is valuable for in-depth analysis and is more suitable for applications requiring a detailed and comprehensive understanding of spectral signatures, especially in remote sensing, environmental monitoring, intelligent agriculture, and national defense.

## 4. Conclusions

The performance difference between NIR technology and HSI using the same 950–1660 nm range spectra for the rapid, non-invasive, and simultaneous evaluation and analysis of WF containing different ADA contents was investigated. For quantitative prediction, the 24 MEWs selected from NIR SG1D spectra using a N_T/V_ = 168/42 dataset and the 23 MEWs from HSI raw spectra using a N_T/V_ = 147/63 dataset enabled the two linear MLR models to quantify ADA with better performance (NIR: R^2^_V_ = 0.9898, RMSE_V_ = 3.06 mg/kg; HSI: R^2^_V_ = 0.9837, RMSE_V_ = 3.86 mg/kg). In addition, based on the 23 MEWs from HSI, color maps were created to achieve the vivid visualization of ADA levels in WF samples. For qualitative discrimination with the same MEWs, the corresponding two MLR-DA models were able to identify WF carrying ADA in excess with 100% accuracy. Both NIR and HSI had a similarly great potential to analyze ADA-contained WF in quantitative and qualitative manners. By contrast, NIR gave a better performance in ADA quantification while HSI provided a direct visualization for ADA observation. From the perspective of industrial applications, NIR technology may be sufficient for the rapid evaluation of WF with different ADA levels.

## Figures and Tables

**Table 1:** Performance of best PLS models for predicting ADA levels (mg/kg) and PLS-DA models for identifying WF samples using full 950–1660 nm spectra from NIR device and from HSI system, based on different numbers of WF samples in training set and validation set, respectively.

NIR Source (N_T/V_ Group)	Spectra	Wavelength Number	Quantitative Analysis (Determine ADA Levels in WF Samples)						Discriminant Analysis (Whether WF Samples Contain Excess ADA)						
			LVs	Training Set		Validation Set			LVs	Training Set			Validation Set		
				R^2^_T_	RMSE_T_	R^2^_V_	RMSE_V_	RPD		R^2^_T_	RMSE_T_	AOC	R^2^_V_	RMSE_V_	AOC
NIR (N_T/V_ = 189/21)	SG1D	355	6	0.9864	3.54	0.9844	3.79	8.0624	6	0.9875	0.11	99.47%	0.9789	0.15	100%
NIR (N_T/V_ = 168/42)	SG1D	355	6	0.9854	3.66	0.9844	3.78	8.0048	6	0.9866	0.12	99.47%	0.9838	0.13	100%
NIR (N_T/V_ = 147/63)	SG1D	355	6	0.9872	3.43	0.9770	4.5	6.6249	6	0.9877	0.11	100%	0.9817	0.14	100%
NIR (N_T/V_ = 126/84)	SG1D	355	6	0.9921	2.69	0.9835	3.88	7.8415	6	0.9924	0.09	100%	0.9898	0.10	100%
NIR (N_T/V_ = 105/105)	SG1D	355	6	0.9896	3.08	0.9825	4.01	7.5584	6	0.9894	0.10	99.47%	0.9786	0.15	100%
HSI-NIR (N_T/V_ = 189/21)	RAW	432	9	0.9850	3.70	0.9872	3.43	8.8350	11	0.9809	0.14	100%	0.9629	0.19	100%
HSI-NIR (N_T/V_ = 168/42)	RAW	432	9	0.9850	3.71	0.9876	3.37	9.0233	11	0.9811	0.14	100%	0.9661	0.18	100%
HSI-NIR (N_T/V_ = 147/63)	RAW	432	9	0.9847	3.75	0.9831	3.94	7.8453	11	0.9832	0.13	100%	0.9584	0.20	100%
HSI-NIR (N_T/V_ = 126/84)	RAW	432	9	0.9856	3.64	0.9812	4.15	7.3107	11	0.9825	0.13	100%	0.9426	0.24	100%
HSI-NIR (N_T/V_ = 105/105)	RAW	432	8	0.9872	3.42	0.9832	3.92	7.8392	10	0.9876	0.11	99.05%	0.9698	0.17	100%

**Table 2:** The most appropriate MEWs selected by SRC method from SG1D spectra (originated from NIR) and raw spectra (originated from HSI), respectively.

NIR Source (N_T/V_ Group)	Preprocessed Spectra	MEWs Selection Method	Number of MEWs	Specific MEWs	Wavelength Reduction
NIR (N_T/V_ = 168/42)	SG1D	SRC	24	989.63, 1009.98, 1016.31, 1044.04, 1047.8, 1052.81, 1156.21, 1173.11, 1206.62, 1240.93, 1242.10, 1281.79, 1295.66, 1335.72, 1343.66, 1407.44, 1443.64, 1445.81, 1468.55, 1480.38, 1485.73, 1513.39, 1570.80, 1608.60	93%
HSI-NIR (N_T/V_ = 147/63)	RAW	SRC	23	1009.192, 1020.709, 1037.16, 1073.349, 1094.732, 1102.955, 1129.27, 1145.717, 1168.743, 1198.349, 1257.572, 1313.525, 1359.627, 1362.921, 1369.509, 1379.393, 1387.63, 1392.573, 1475.006, 1557.564, 1577.399, 1593.936, 1633.652	95%

**Table 3:** Best results of predicting ADA levels in WF using MEWs based on the different numbers of WF samples in training set and validation set.

NIR Source (N_T/V_ Group)	Spectra	Number of MEWs	Modeling Algorithm	Quantitative Regression Analysis						Discriminant Analysis (Whether Excessive ADA)						
				LVs	Training Set		Validation Set			LVs	Training Set			Validation Set		
					R^2^_T_	RMSE_T_	R^2^_V_	RMSE_V_	RPD		R^2^_T_	RMSE_T_	AOC	R^2^_V_	RMSE_V_	AOC
NIR (N_T/V_ = 168/42)	SG1D	24	PLS	4	0.9810	4.18	0.9819	4.08	7.4889	3	0.9091	0.30	98.21%	0.9119	0.30	97.62%
			MLR	-	0.9914	2.81	0.9898	3.06	9.9001	-	0.9410	0.24	99.40%	0.9369	0.25	100%
HSI-NIR (N_T/V_ = 147/63)	RAW	23	PLS	7	0.9892	3.15	0.9835	3.88	7.7972	8	0.9038	0.31	98.64%	0.8765	0.35	100%
			MLR	-	0.9919	2.72	0.9837	3.86	7.8388	-	0.9101	0.30	99.32%	0.8787	0.35	100%

## Data Availability

The original contributions presented in this study are included in the article/Supplementary Materials. Further inquiries can be directed to the corresponding author.

## Supplementary Materials

The following supporting information can be downloaded at: https://www.mdpi.com/article/10.3390/foods13223667/s1, Figure S1: The preprocessed spectral features of all WF samples acquired from the NIR device (a) and HSI system (b); Table S1: Performance of PLS models for predicting ADA levels (mg/kg) and PLS-DA models for identifying WF samples using full 950–1660 nm spectra from the NIR device and from the HSI system, based on different numbers of WF samples in training set and validation set, respectively; Table S2: The MEWs selected by SRC method from SG1D spectra (originated from NIR) and raw spectra (originated from HSI), respectively; Table S3: Predicting ADA levels in WF using MEWs based on the different numbers of WF samples in training set and validation set.
