# Supplementary material for: Comparative Quantitative and Discriminant Analysis of Wheat Flour with Different Levels of Chemical Azodicarbonamide Using NIR Spectroscopy and Hyperspectral Imaging
Source: Foods. 2024 Nov 18;13(22):3667. doi: 10.3390/foods13223667 (PMC11593926; doi:10.3390/foods13223667)
Supplement: Supplementary file 1 [file foods-13-03667-s001.zip › foods-3251856-supplementary.pdf]

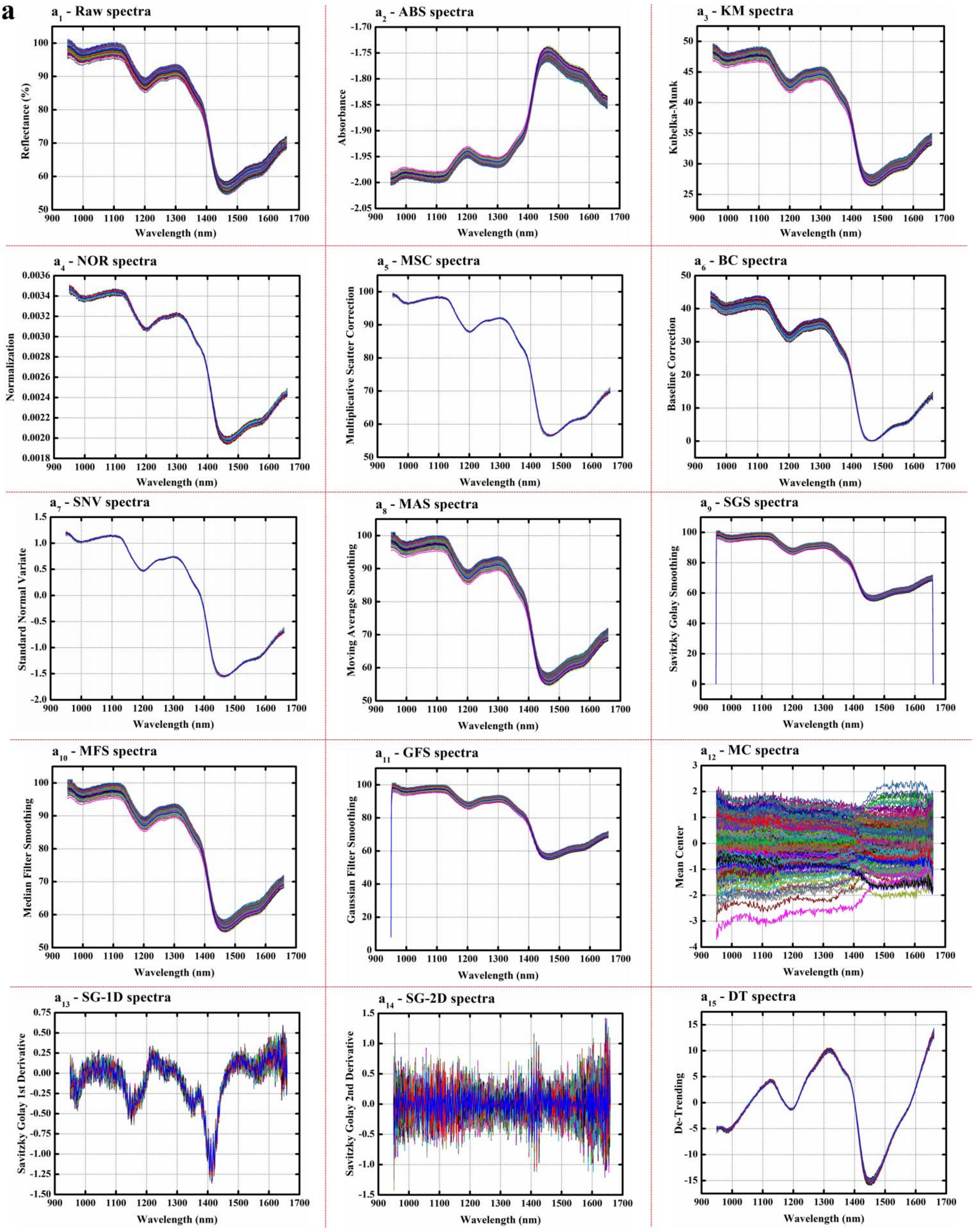

a
$a_1$ - Raw spectra
Reflectance (%)
Wavelength (nm)
$a_2$ - ABS spectra
Absorbance
Wavelength (nm)
$a_3$ - KM spectra
Kubelka-Munk
Wavelength (nm)
$a_4$ - NOR spectra
Normalization
Wavelength (nm)
$a_5$ - MSC spectra
Multiplicative Scatter Correction
Wavelength (nm)
$a_6$ - BC spectra
Baseline Correction
Wavelength (nm)
$a_7$ - SNV spectra
Standard Normal Variate
Wavelength (nm)
$a_8$ - MAS spectra
Moving Average Smoothing
Wavelength (nm)
$a_9$ - SGS spectra
Savitzky Golay Smoothing
Wavelength (nm)
$a_{10}$ - MFS spectra
Median Filter Smoothing
Wavelength (nm)
$a_{11}$ - GFS spectra
Gaussian Filter Smoothing
Wavelength (nm)
$a_{12}$ - MC spectra
Mean Center
Wavelength (nm)
$a_{13}$ - SG-1D spectra
Savitzky Golay 1st Derivative
Wavelength (nm)
$a_{14}$ - SG-2D spectra
Savitzky Golay 2nd Derivative
Wavelength (nm)
$a_{15}$ - DT spectra
De-Trending
Wavelength (nm)

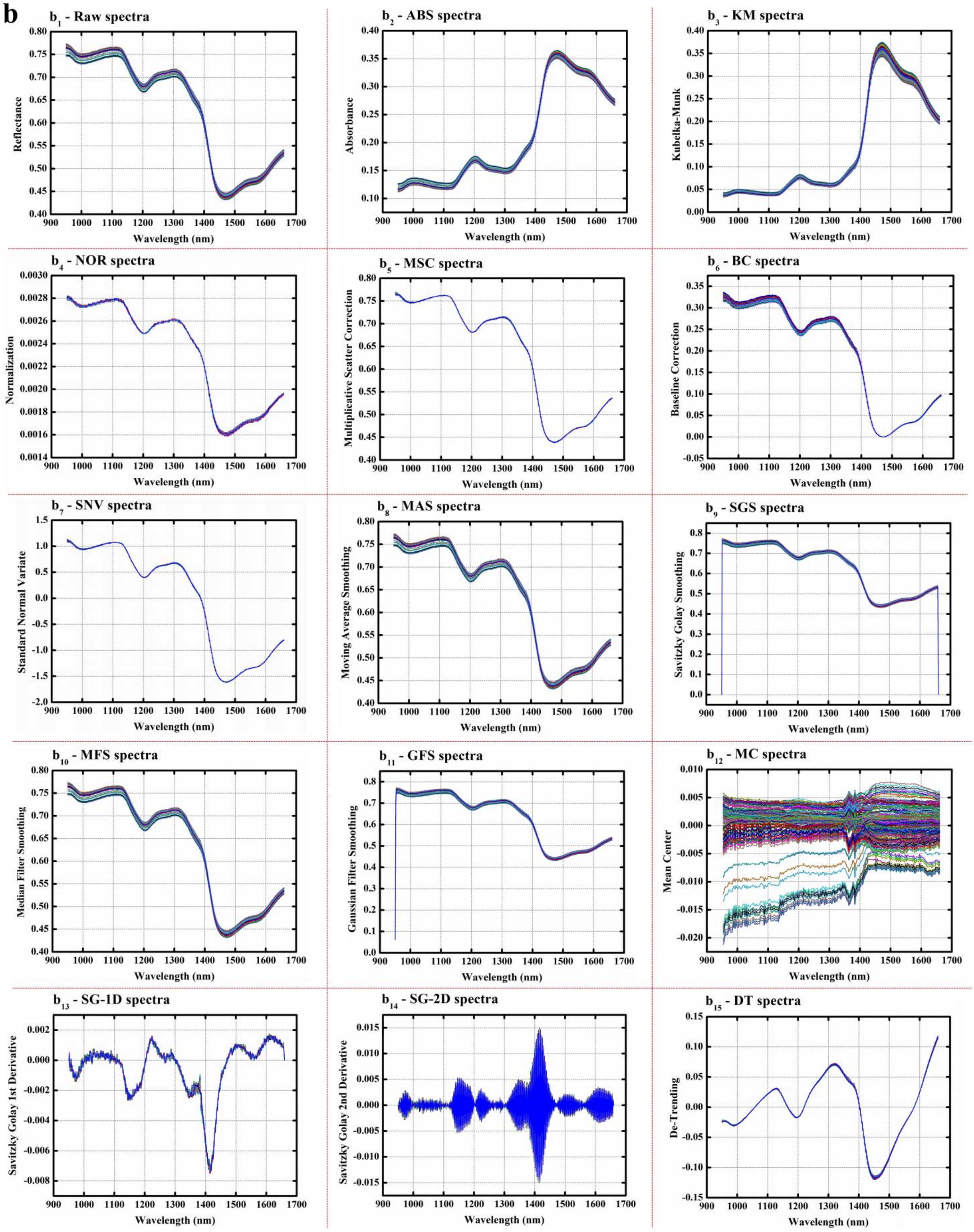


**Figure S1.** The preprocessed spectral features of all WF samples acquired from NIR device (a) and HSI system (b), respectively.

**Table S1:** Performances of PLS models for predicting ADA levels (mg/kg) and PLS-DA models for identifying WF samples using full 950–1660 nm spectra from NIR device and from HSI system, based on different numbers of WF samples in training set and validation set, respectively.

| NIR source ($N_{T/V}$ group) | Preprocessed spectra | Number of wavelengths | Modeling algorithm | Quantitative Analysis (determine ADA levels in WF samples) | | | | | | | Discriminant Analysis (whether WF samples contain excess ADA) | | | | | | |
|---|---|---|---|---|---|---|---|---|---|---|---|---|---|---|---|---|---|
| | | | | LVs | Training set | | Validation set | | | | LVs | Training set | | | Validation set | | |
| | | | | | $R^2_T$ | $RMSE_T$ | $R^2_V$ | $RMSE_V$ | RPD | Bias | | $R^2_T$ | $RMSE_T$ | AOC | $R^2_V$ | $RMSE_V$ | AOC |
| NIR ($N_{T/V}$=189/21) | RAW | 355 | PLS | 8 | 0.9823 | 4.03 | 0.9737 | 4.91 | 6.1754 | -0.1891 | 8 | 0.9842 | 0.13 | 99.47% | 0.9698 | 0.17 | 100% |
| | ABS | 355 | PLS | 8 | 0.9840 | 3.83 | 0.9758 | 4.72 | 6.4310 | -0.2524 | 8 | 0.9864 | 0.12 | 99.47% | 0.9785 | 0.15 | 100% |
| | KM | 355 | PLS | 8 | 0.9823 | 4.03 | 0.9737 | 4.91 | 6.1753 | -0.1891 | 8 | 0.9842 | 0.13 | 99.47% | 0.9698 | 0.17 | 100% |
| | NOR | 355 | PLS | 8 | 0.9864 | 3.53 | 0.9782 | 4.47 | 6.7739 | -0.1898 | 7 | 0.9828 | 0.13 | 99.47% | 0.9706 | 0.18 | 100% |
| | MSC | 355 | PLS | 8 | 0.9909 | 2.89 | 0.9866 | 3.50 | 8.6559 | -0.1106 | 8 | 0.9907 | 0.096 | 100% | 0.9828 | 0.13 | 100% |
| | BC | 355 | PLS | 8 | 0.9874 | 3.40 | 0.9818 | 4.08 | 7.4621 | -0.2245 | 8 | 0.9892 | 0.10 | 100% | 0.9811 | 0.14 | 100% |
| | SNV | 355 | PLS | 8 | 0.9909 | 2.89 | 0.9866 | 3.50 | 8.6556 | -0.1109 | 8 | 0.9907 | 0.096 | 100% | 0.9828 | 0.13 | 100% |
| | MAS | 355 | PLS | 10 | 0.9883 | 3.28 | 0.9845 | 3.77 | 8.0253 | 0.1305 | 10 | 0.9887 | 0.11 | 100% | 0.9854 | 0.12 | 100% |
| | SGS | 355 | PLS | 10 | 0.9887 | 3.22 | 0.9858 | 3.60 | 8.4093 | 0.1423 | 10 | 0.9886 | 0.11 | 100% | 0.9843 | 0.13 | 100% |
| | MFS | 355 | PLS | 10 | 0.9908 | 2.90 | 0.9896 | 3.08 | 9.8251 | -0.08181 | 10 | 0.9895 | 0.10 | 100% | 0.9879 | 0.11 | 100% |
| | GFS | 355 | PLS | 10 | 0.9953 | 2.08 | 0.9933 | 2.47 | 12.2907 | -0.2333 | 9 | 0.9878 | 0.11 | 99.47% | 0.9805 | 0.14 | 100% |
| | MC | 355 | PLS | 8 | 0.9822 | 4.04 | 0.9736 | 4.92 | 6.1558 | -0.09857 | 8 | 0.9842 | 0.13 | 99.47% | 0.9698 | 0.17 | 100% |
| | **SG1D** | **355** | **PLS** | **6** | **0.9864** | **3.54** | **0.9844** | **3.79** | **8.0624** | **0.4845** | **6** | **0.9875** | **0.11** | **99.47%** | **0.9789** | **0.15** | **100%** |
| | SG2D | 355 | PLS | 5 | 0.9783 | 4.46 | 0.9703 | 5.22 | 5.8644 | -0.7775 | 6 | 0.9864 | 0.12 | 100% | 0.9827 | 0.13 | 100% |
| | DT | 355 | PLS | 8 | 0.9916 | 2.77 | 0.9870 | 3.45 | 8.7941 | 0.1494 | 7 | 0.9864 | 0.12 | 100% | 0.9763 | 0.15 | 100% |
| NIR ($N_{T/V}$=168/42) | RAW | 355 | PLS | 8 | 0.9821 | 4.05 | 0.9812 | 4.15 | 7.3018 | 0.1084 | 8 | 0.9841 | 0.13 | 99.47% | 0.9799 | 0.14 | 100% |
| | ABS | 355 | PLS | 8 | 0.9834 | 3.90 | 0.9808 | 4.19 | 7.2218 | 0.1455 | 8 | 0.9862 | 0.12 | 99.47% | 0.9838 | 0.13 | 100% |
| | KM | 355 | PLS | 8 | 0.9821 | 4.05 | 0.9812 | 4.15 | 7.3018 | 0.1079 | 8 | 0.9841 | 0.13 | 99.47% | 0.9799 | 0.14 | 100% |
| | NOR | 355 | PLS | 8 | 0.9864 | 3.53 | 0.9849 | 3.72 | 8.1416 | 0.1554 | 8 | 0.9883 | 0.11 | 99.47% | 0.9849 | 0.12 | 100% |
| | MSC | 355 | PLS | 8 | 0.9907 | 2.92 | 0.9891 | 3.16 | 9.5881 | 0.0115 | 8 | 0.9907 | 0.096 | 99.47% | 0.9875 | 0.11 | 100% |
| | BC | 355 | PLS | 8 | 0.9871 | 3.43 | 0.9858 | 3.61 | 8.4095 | 0.2030 | 8 | 0.9893 | 0.10 | 99.47% | 0.9864 | 0.12 | 100% |
| | SNV | 355 | PLS | 8 | 0.9907 | 2.92 | 0.9891 | 3.16 | 9.5874 | 0.01191 | 8 | 0.9907 | 0.096 | 99.47% | 0.9875 | 0.11 | 100% |
| | MAS | 355 | PLS | 10 | 0.9883 | 3.27 | 0.9870 | 3.45 | 8.8342 | 0.3659 | 10 | 0.9888 | 0.11 | 100% | 0.9878 | 0.11 | 100% |
| | SGS | 355 | PLS | 10 | 0.9888 | 3.20 | 0.9881 | 3.31 | 9.2066 | 0.3523 | 10 | 0.9887 | 0.11 | 100% | 0.9872 | 0.11 | 100% |
| | MFS | 355 | PLS | 10 | 0.9902 | 3.00 | 0.9904 | 2.97 | 10.2025 | 0.06437 | 10 | 0.9891 | 0.10 | 99.47% | 0.9894 | 0.10 | 100% |
| | GFS | 355 | PLS | 10 | 0.9950 | 2.13 | 0.9943 | 2.28 | 13.2921 | -0.1364 | 10 | 0.9937 | 0.079 | 100% | 0.9924 | 0.087 | 100% |
| | MC | 355 | PLS | 8 | 0.9821 | 4.05 | 0.9812 | 4.15 | 7.3018 | 0.1078 | 8 | 0.9841 | 0.13 | 99.47% | 0.9799 | 0.14 | 100% |
| | **SG1D** | **355** | **PLS** | **6** | **0.9854** | **3.66** | **0.9844** | **3.78** | **8.0048** | **0.07787** | **6** | **0.9866** | **0.12** | **99.47%** | **0.9838** | **0.13** | **100%** |
| | SG2D | 355 | PLS | 5 | 0.9778 | 4.51 | 0.9755 | 4.74 | 6.4193 | -0.4186 | 5 | 0.9766 | 0.15 | 99.47% | 0.9730 | 0.16 | 100% |
| | DT | 355 | PLS | 7 | 0.9861 | 3.58 | 0.9858 | 3.61 | 8.3900 | -0.09138 | 7 | 0.9831 | 0.13 | 100% | 0.9809 | 0.14 | 100% |
| NIR ($N_{T/V}$=147/63) | RAW | 355 | PLS | 8 | 0.9849 | 3.72 | 0.9716 | 5.10 | 5.9333 | 0.1161 | 8 | 0.9837 | 0.13 | 100% | 0.9692 | 0.18 | 100% |
| | ABS | 355 | PLS | 8 | 0.9846 | 3.76 | 0.9725 | 5.02 | 6.0287 | 0.1442 | 8 | 0.9842 | 0.13 | 100% | 0.9690 | 0.18 | 100% |
| | KM | 355 | PLS | 8 | 0.9849 | 3.72 | 0.9716 | 5.10 | 5.9332 | 0.1160 | 8 | 0.9837 | 0.13 | 100% | 0.9692 | 0.18 | 100% |
| | NOR | 355 | PLS | 7 | 0.9828 | 3.97 | 0.9703 | 5.22 | 5.8028 | 0.09055 | 8 | 0.9878 | 0.11 | 100% | 0.9764 | 0.15 | 100% |
| | MSC | 355 | PLS | 6 | 0.9734 | 4.93 | 0.9621 | 5.90 | 5.1349 | -0.04846 | 7 | 0.9863 | 0.12 | 99.47% | 0.9778 | 0.15 | 100% |
| | BC | 355 | PLS | 8 | 0.9886 | 3.24 | 0.9730 | 4.97 | 6.0900 | 0.1450 | 8 | 0.9889 | 0.11 | 100% | 0.9778 | 0.15 | 100% |

| | | | | | | | | | | | | | | | | | |
|---|---|---|---|---|---|---|---|---|---|---|---|---|---|---|---|---|---|
| | SNV | 355 | PLS | 6 | 0.9734 | 4.93 | 0.9621 | 5.90 | 5.1346 | -0.04866 | 7 | 0.9863 | 0.12 | 99.47% | 0.9778 | 0.15 | 100% |
| | MAS | 355 | PLS | 10 | 0.9882 | 3.29 | 0.9677 | 5.44 | 5.5726 | 0.2368 | 10 | 0.9886 | 0.11 | 100% | 0.9779 | 0.15 | 100% |
| | SGS | 355 | PLS | 10 | 0.9883 | 3.28 | 0.9672 | 5.48 | 5.5328 | 0.2780 | 10 | 0.9884 | 0.11 | 100% | 0.9779 | 0.15 | 100% |
| | MFS | 355 | PLS | 10 | 0.9909 | 2.89 | 0.9703 | 5.22 | 5.8030 | 0.1252 | 10 | 0.9898 | 0.10 | 100% | 0.9765 | 0.15 | 100% |
| | GFS | 355 | PLS | 9 | 0.9895 | 3.10 | 0.9759 | 4.70 | 6.4618 | 0.3947 | 10 | 0.9942 | 0.08 | 100% | 0.9849 | 0.12 | 100% |
| | MC | 355 | PLS | 8 | 0.9849 | 3.72 | 0.9716 | 5.10 | 5.9333 | 0.1162 | 8 | 0.9837 | 0.13 | 100% | 0.9692 | 0.18 | 100% |
| | **SG1D** | **355** | **PLS** | **6** | **0.9872** | **3.43** | **0.9770** | **4.59** | **6.6249** | **0.4254** | **6** | **0.9877** | **0.11** | **100%** | **0.9817** | **0.14** | **100%** |
| | SG2D | 355 | PLS | 6 | 0.9875 | 3.38 | 0.9729 | 4.99 | 6.0902 | 0.4144 | 5 | 0.9750 | 0.16 | 99.47% | 0.9641 | 0.19 | 100% |
| | DT | 355 | PLS | 7 | 0.9865 | 3.52 | 0.9627 | 5.85 | 5.1925 | 0.4766 | 8 | 0.9905 | 0.10 | 100% | 0.9767 | 0.15 | 100% |
| NIR ($N_{T/V}$=126/84) | RAW | 355 | PLS | 8 | 0.9845 | 3.77 | 0.9775 | 4.54 | 6.6686 | -0.1240 | 8 | 0.9868 | 0.11 | 100% | 0.9727 | 0.17 | 100% |
| | ABS | 355 | PLS | 8 | 0.9853 | 3.67 | 0.9825 | 4.01 | 7.5585 | 0.05203 | 8 | 0.9887 | 0.11 | 100% | 0.9728 | 0.16 | 100% |
| | KM | 355 | PLS | 8 | 0.9845 | 3.77 | 0.9775 | 4.54 | 6.6683 | -0.1238 | 8 | 0.9868 | 0.11 | 100% | 0.9727 | 0.17 | 100% |
| | NOR | 355 | PLS | 8 | 0.9921 | 2.69 | 0.9821 | 4.05 | 7.4991 | -0.2808 | 8 | 0.9925 | 0.09 | 100% | 0.9751 | 0.16 | 100% |
| | MSC | 355 | PLS | 8 | 0.9922 | 2.68 | 0.9790 | 4.39 | 6.9510 | -0.5660 | 8 | 0.9923 | 0.09 | 100% | 0.9821 | 0.13 | 100% |
| | BC | 355 | PLS | 8 | 0.9899 | 3.05 | 0.9814 | 4.13 | 7.3834 | -0.5114 | 8 | 0.9914 | 0.09 | 100% | 0.9772 | 0.15 | 100% |
| | SNV | 355 | PLS | 8 | 0.9922 | 2.68 | 0.9790 | 4.39 | 6.9511 | -0.5663 | 8 | 0.9923 | 0.09 | 100% | 0.9821 | 0.13 | 100% |
| | MAS | 355 | PLS | 10 | 0.9890 | 3.17 | 0.9841 | 3.82 | 7.9743 | -0.4212 | 10 | 0.9887 | 0.11 | 100% | 0.9679 | 0.16 | 100% |
| | SGS | 355 | PLS | 10 | 0.9896 | 3.09 | 0.9856 | 3.64 | 8.3944 | -0.4758 | 10 | 0.9888 | 0.11 | 100% | 0.9762 | 0.15 | 100% |
| | MFS | 355 | PLS | 10 | 0.9915 | 2.79 | 0.9826 | 4.00 | 7.5762 | -0.01752 | 10 | 0.9887 | 0.11 | 100% | 0.9746 | 0.16 | 100% |
| | GFS | 355 | PLS | 10 | 0.9948 | 2.19 | 0.9859 | 3.59 | 8.5096 | -0.4838 | 9 | 0.9895 | 0.10 | 100% | 0.9713 | 0.17 | 100% |
| | MC | 355 | PLS | 8 | 0.9845 | 3.77 | 0.9775 | 4.54 | 6.6686 | -0.1237 | 8 | 0.9868 | 0.11 | 100% | 0.9727 | 0.17 | 100% |
| | **SG1D** | **355** | **PLS** | **6** | **0.9921** | **2.69** | **0.9835** | **3.88** | **7.8415** | **-0.4188** | **6** | **0.9924** | **0.09** | **100%** | **0.9898** | **0.10** | **100%** |
| | SG2D | 355 | PLS | 5 | 0.9800 | 4.28 | 0.9654 | 5.63 | 5.3773 | -0.1017 | 6 | 0.9874 | 0.11 | 100% | 0.9800 | 0.14 | 100% |
| | DT | 355 | PLS | 8 | 0.9937 | 2.40 | 0.9882 | 3.29 | 9.2508 | -0.2911 | 7 | 0.9884 | 0.11 | 100% | 0.9727 | 0.17 | 100% |
| NIR ($N_{T/V}$=105/105) | RAW | 355 | PLS | 8 | 0.9846 | 3.76 | 0.9692 | 5.31 | 5.7103 | -0.2922 | 8 | 0.9838 | 0.13 | 99.47% | 0.9697 | 0.17 | 100% |
| | ABS | 355 | PLS | 8 | 0.9867 | 3.50 | 0.9737 | 4.91 | 6.1879 | -0.3982 | 8 | 0.9856 | 0.12 | 99.47% | 0.9823 | 0.13 | 100% |
| | KM | 355 | PLS | 8 | 0.9846 | 3.76 | 0.9692 | 5.31 | 5.7101 | -0.2922 | 8 | 0.9838 | 0.13 | 99.47% | 0.9697 | 0.17 | 100% |
| | NOR | 355 | PLS | 6 | 0.9710 | 5.16 | 0.9511 | 6.69 | 4.5312 | -0.3932 | 7 | 0.9823 | 0.13 | 99.47% | 0.9675 | 0.18 | 100% |
| | MSC | 355 | PLS | 8 | 0.9905 | 2.95 | 0.9808 | 4.19 | 7.2314 | -0.2443 | 8 | 0.9901 | 0.10 | 99.47% | 0.9804 | 0.14 | 100% |
| | BC | 355 | PLS | 7 | 0.9828 | 3.97 | 0.9657 | 5.61 | 5.4123 | -0.3767 | 7 | 0.9808 | 0.14 | 99.47% | 0.9851 | 0.12 | 100% |
| | SNV | 355 | PLS | 8 | 0.9905 | 2.95 | 0.9808 | 4.19 | 7.2315 | -0.2446 | 8 | 0.9901 | 0.10 | 99.47% | 0.9804 | 0.14 | 100% |
| | MAS | 355 | PLS | 9 | 0.9800 | 4.28 | 0.9570 | 6.28 | 4.8383 | -0.5383 | 11 | 0.9939 | 0.08 | 99.47% | 0.9881 | 0.11 | 100% |
| | SGS | 355 | PLS | 7 | 0.9635 | 5.78 | 0.9439 | 7.17 | 4.2280 | -0.4095 | 11 | 0.9941 | 0.08 | 99.47% | 0.9882 | 0.11 | 100% |
| | MFS | 355 | PLS | 7 | 0.9621 | 5.90 | 0.9446 | 7.13 | 4.2591 | -0.4876 | 11 | 0.9955 | 0.07 | 99.47% | 0.9897 | 0.10 | 100% |
| | GFS | 355 | PLS | 8 | 0.9824 | 4.02 | 0.9667 | 5.53 | 5.4911 | -0.3732 | 10 | 0.9929 | 0.08 | 99.47% | 0.9860 | 0.12 | 100% |
| | MC | 355 | PLS | 8 | 0.9848 | 3.73 | 0.9705 | 5.20 | 5.8330 | -0.2483 | 8 | 0.9838 | 0.13 | 99.47% | 0.9697 | 0.17 | 100% |
| | **SG1D** | **355** | **PLS** | **6** | **0.9896** | **3.08** | **0.9825** | **4.01** | **7.5584** | **0.1097** | **6** | **0.9894** | **0.10** | **99.47%** | **0.9786** | **0.15** | **100%** |
| | SG2D | 355 | PLS | 5 | 0.9790 | 4.39 | 0.9635 | 5.79 | 5.2385 | -0.2815 | 5 | 0.9797 | 0.14 | 99.47% | 0.9605 | 0.20 | 100% |
| | DT | 355 | PLS | 8 | 0.9927 | 2.59 | 0.9862 | 3.56 | 8.5107 | -0.1768 | 7 | 0.9870 | 0.11 | 99.47% | 0.9754 | 0.16 | 100% |
| HSI-NIR ($N_{T/V}$=189/21) | **RAW** | **432** | **PLS** | **9** | **0.9850** | **3.70** | **0.9872** | **3.43** | **8.8350** | **-0.1358** | **11** | **0.9809** | **0.14** | **100%** | **0.9629** | **0.19** | **100%** |
| | ABS | 432 | PLS | 9 | 0.9832 | 3.92 | 0.9836 | 3.87 | 7.8201 | -0.08105 | 11 | 0.9797 | 0.14 | 100% | 0.9584 | 0.20 | 100% |
| | KM | 432 | PLS | 9 | 0.9843 | 3.80 | 0.9796 | 4.32 | 7.0308 | -0.3899 | 11 | 0.9692 | 0.18 | 100% | 0.9359 | 0.25 | 100% |
| | NOR | 432 | PLS | 8 | 0.9820 | 4.07 | 0.9802 | 4.26 | 7.1594 | 0.5126 | 11 | 0.9832 | 0.13 | 100% | 0.9628 | 0.19 | 100% |
| | MSC | 432 | PLS | 7 | 0.9784 | 4.45 | 0.9744 | 4.85 | 6.2492 | -0.1931 | 10 | 0.9825 | 0.13 | 100% | 0.9627 | 0.19 | 100% |

| | | | | | | | | | | | | | | | | | |
|---|---|---|---|---|---|---|---|---|---|---|---|---|---|---|---|---|---|
| | BC | 432 | PLS | 9 | 0.9897 | 3.07 | 0.9867 | 3.49 | 8.6689 | -0.05602 | 11 | 0.9763 | 0.15 | 100% | 0.9503 | 0.22 | 100% |
| | SNV | 432 | PLS | 7 | 0.9784 | 4.45 | 0.9743 | 4.85 | 6.2487 | -0.1929 | 10 | 0.9825 | 0.13 | 100% | 0.9627 | 0.19 | 100% |
| | MAS | 432 | PLS | 9 | 0.9824 | 4.01 | 0.9845 | 3.76 | 8.0486 | -0.1124 | 11 | 0.9712 | 0.17 | 100% | 0.9403 | 0.24 | 100% |
| | SGS | 432 | PLS | 9 | 0.9826 | 3.99 | 0.9845 | 3.77 | 8.0398 | -0.1334 | 11 | 0.9719 | 0.17 | 100% | 0.9399 | 0.24 | 100% |
| | MFS | 432 | PLS | 9 | 0.9839 | 3.84 | 0.9858 | 3.61 | 8.4089 | -0.2015 | 11 | 0.9767 | 0.15 | 100% | 0.9507 | 0.22 | 100% |
| | GFS | 432 | PLS | 9 | 0.9843 | 3.80 | 0.9863 | 3.54 | 8.5676 | -0.1603 | 11 | 0.9797 | 0.14 | 100% | 0.9589 | 0.20 | 100% |
| | MC | 432 | PLS | 9 | 0.9850 | 3.70 | 0.9872 | 3.43 | 8.8350 | -0.1356 | 11 | 0.9809 | 0.14 | 100% | 0.9629 | 0.19 | 100% |
| | SG1D | 432 | PLS | 6 | 0.9768 | 4.62 | 0.9712 | 5.13 | 5.9028 | -0.07497 | 6 | 0.9538 | 0.21 | 100% | 0.9250 | 0.27 | 100% |
| | SG2D | 432 | PLS | 6 | 0.9771 | 4.58 | 0.9734 | 4.94 | 6.1333 | 0.03004 | 6 | 0.9532 | 0.22 | 100% | 0.9309 | 0.26 | 100% |
| | DT | 432 | PLS | 6 | 0.9668 | 5.52 | 0.9601 | 6.05 | 5.0104 | 0.1992 | 10 | 0.9785 | 0.15 | 100% | 0.9485 | 0.23 | 100% |
| HSI-NIR ($N_{T/V}$=168/42) | **RAW** | **432** | **PLS** | **9** | **0.9850** | **3.71** | **0.9876** | **3.37** | **9.0233** | **0.3178** | **11** | **0.9811** | **0.14** | **100%** | **0.9661** | **0.18** | **100%** |
| | ABS | 432 | PLS | 9 | 0.9830 | 3.94 | 0.9851 | 3.69 | 8.2217 | 0.2874 | 11 | 0.9805 | 0.14 | 100% | 0.9709 | 0.17 | 100% |
| | KM | 432 | PLS | 9 | 0.9839 | 3.85 | 0.9832 | 3.92 | 7.7332 | -0.2408 | 11 | 0.9703 | 0.17 | 100% | 0.9417 | 0.24 | 100% |
| | NOR | 432 | PLS | 8 | 0.9811 | 4.16 | 0.9822 | 4.04 | 7.5813 | 0.6350 | 11 | 0.9836 | 0.13 | 100% | 0.9662 | 0.18 | 100% |
| | MSC | 432 | PLS | 8 | 0.9842 | 3.81 | 0.9848 | 3.74 | 8.1100 | 0.1688 | 10 | 0.9833 | 0.13 | 100% | 0.9644 | 0.19 | 100% |
| | BC | 432 | PLS | 8 | 0.9799 | 4.29 | 0.9816 | 4.11 | 7.4746 | 0.6931 | 11 | 0.9765 | 0.15 | 99.40% | 0.9689 | 0.18 | 100% |
| | SNV | 432 | PLS | 8 | 0.9842 | 3.81 | 0.9848 | 3.74 | 8.1100 | 0.1684 | 10 | 0.9833 | 0.13 | 100% | 0.9644 | 0.19 | 100% |
| | MAS | 432 | PLS | 9 | 0.9820 | 4.06 | 0.9856 | 3.63 | 8.4125 | 0.4613 | 11 | 0.9709 | 0.17 | 99.40% | 0.9557 | 0.21 | 100% |
| | SGS | 432 | PLS | 9 | 0.9823 | 4.03 | 0.9858 | 3.61 | 8.4620 | 0.4520 | 11 | 0.9717 | 0.17 | 99.40% | 0.9553 | 0.21 | 100% |
| | MFS | 432 | PLS | 9 | 0.9834 | 3.90 | 0.9867 | 3.50 | 8.6980 | 0.3338 | 11 | 0.9767 | 0.15 | 100% | 0.9607 | 0.20 | 100% |
| | GFS | 432 | PLS | 9 | 0.9841 | 3.82 | 0.9872 | 3.43 | 8.8796 | 0.3298 | 11 | 0.9796 | 0.14 | 100% | 0.9648 | 0.19 | 100% |
| | MC | 432 | PLS | 9 | 0.9850 | 3.71 | 0.9876 | 3.37 | 9.0233 | 0.3177 | 11 | 0.9811 | 0.14 | 100% | 0.9661 | 0.18 | 100% |
| | SG1D | 432 | PLS | 6 | 0.9758 | 4.71 | 0.9664 | 5.55 | 5.4569 | -0.1073 | 6 | 0.9552 | 0.21 | 99.40% | 0.9218 | 0.28 | 100% |
| | SG2D | 432 | PLS | 6 | 0.9763 | 4.66 | 0.9671 | 5.50 | 5.5097 | -0.05720 | 6 | 0.9554 | 0.21 | 100% | 0.9198 | 0.28 | 100% |
| | DT | 432 | PLS | 6 | 0.9658 | 5.60 | 0.9684 | 5.38 | 5.6561 | 0.5637 | 10 | 0.9786 | 0.15 | 99.40% | 0.9592 | 0.20 | 100% |
| HSI-NIR ($N_{T/V}$=147/63) | **RAW** | **432** | **PLS** | **9** | **0.9847** | **3.75** | **0.9831** | **3.94** | **7.8453** | **0.7764** | **11** | **0.9832** | **0.13** | **100%** | **0.9584** | **0.20** | **100%** |
| | ABS | 432 | PLS | 9 | 0.9826 | 3.99 | 0.9817 | 4.10 | 7.5451 | 0.8256 | 11 | 0.9823 | 0.13 | 99.32% | 0.9785 | 0.15 | 100% |
| | KM | 432 | PLS | 9 | 0.9823 | 4.03 | 0.9790 | 4.39 | 7.2530 | 1.3518 | 11 | 0.9714 | 0.17 | 99.32% | 0.9410 | 0.24 | 99.32% |
| | NOR | 432 | PLS | 9 | 0.9829 | 3.96 | 0.9842 | 3.81 | 8.0341 | 0.5295 | 11 | 0.9857 | 0.12 | 100% | 0.9597 | 0.20 | 100% |
| | MSC | 432 | PLS | 7 | 0.9850 | 3.71 | 0.9834 | 3.90 | 7.7958 | 0.3399 | 10 | 0.9847 | 0.12 | 100% | 0.9593 | 0.20 | 100% |
| | BC | 432 | PLS | 8 | 0.9807 | 4.20 | 0.9824 | 4.01 | 7.5851 | 0.4199 | 12 | 0.9872 | 0.11 | 100% | 0.9615 | 0.20 | 100% |
| | SNV | 432 | PLS | 7 | 0.9850 | 3.71 | 0.9834 | 3.90 | 7.7958 | 0.3398 | 10 | 0.9847 | 0.12 | 100% | 0.9593 | 0.20 | 100% |
| | MAS | 432 | PLS | 9 | 0.9829 | 3.96 | 0.9816 | 4.11 | 7.4460 | 0.6155 | 11 | 0.9742 | 0.16 | 99.32% | 0.9423 | 0.24 | 100% |
| | SGS | 432 | PLS | 9 | 0.9831 | 3.94 | 0.9817 | 4.10 | 7.4690 | 0.5971 | 11 | 0.9751 | 0.16 | 99.32% | 0.9439 | 0.24 | 100% |
| | MFS | 432 | PLS | 9 | 0.9838 | 3.86 | 0.9829 | 3.96 | 7.7704 | 0.6996 | 11 | 0.9788 | 0.15 | 99.32% | 0.9483 | 0.23 | 100% |
| | GFS | 432 | PLS | 9 | 0.9841 | 3.82 | 0.9824 | 4.01 | 3.9814 | 0.7162 | 11 | 0.9819 | 0.13 | 100% | 0.9557 | 0.21 | 100% |
| | MC | 432 | PLS | 9 | 0.9847 | 3.75 | 0.9831 | 3.94 | 7.8453 | 0.7769 | 11 | 0.9832 | 0.13 | 100% | 0.9584 | 0.20 | 100% |
| | SG1D | 432 | PLS | 6 | 0.9778 | 4.51 | 0.9709 | 5.16 | 6.0000 | 1.0997 | 6 | 0.9503 | 0.22 | 100% | 0.9343 | 0.26 | 100% |
| | SG2D | 432 | PLS | 6 | 0.9782 | 4.47 | 0.9715 | 5.11 | 6.1020 | 1.2128 | 7 | 0.9659 | 0.18 | 100% | 0.9438 | 0.24 | 100% |
| | DT | 432 | PLS | 6 | 0.9660 | 5.59 | 0.9681 | 5.41 | 5.6013 | 0.1717 | 11 | 0.9901 | 0.10 | 100% | 0.9584 | 0.20 | 100% |
| HSI-NIR ($N_{T/V}$=126/84) | **RAW** | **432** | **PLS** | **9** | **0.9856** | **3.64** | **0.9812** | **4.15** | **7.3107** | **0.2519** | **11** | **0.9825** | **0.13** | **100%** | **0.9426** | **0.24** | **100%** |
| | ABS | 432 | PLS | 10 | 0.9927 | 2.58 | 0.9869 | 3.46 | 8.7585 | 0.1604 | 11 | 0.9812 | 0.14 | 100% | 0.9721 | 0.17 | 100% |
| | KM | 432 | PLS | 9 | 0.9842 | 3.81 | 0.9758 | 4.71 | 6.4954 | 0.7001 | 10 | 0.9636 | 0.19 | 100% | 0.9049 | 0.31 | 100% |
| | NOR | 432 | PLS | 8 | 0.9814 | 4.13 | 0.9759 | 4.70 | 6.4604 | 0.2975 | 10 | 0.9748 | 0.16 | 99.21% | 0.9256 | 0.27 | 100% |

| | | | | | | | | | | | | | | | | | |
|---|---|---|---|---|---|---|---|---|---|---|---|---|---|---|---|---|---|
| | MSC | 432 | PLS | 8 | 0.9868 | 3.48 | 0.9779 | 4.50 | 6.7612 | 0.4539 | 10 | 0.9835 | 0.13 | 100% | 0.9429 | 0.24 | 100% |
| | BC | 432 | PLS | 8 | 0.9802 | 4.26 | 0.9748 | 4.80 | 6.3140 | 0.3029 | 10 | 0.9720 | 0.17 | 99.21% | 0.9314 | 0.26 | 100% |
| | SNV | 432 | PLS | 8 | 0.9868 | 3.48 | 0.9779 | 4.50 | 6.7617 | 0.4548 | 10 | 0.9835 | 0.13 | 100% | 0.9429 | 0.24 | 100% |
| | MAS | 432 | PLS | 9 | 0.9825 | 4.01 | 0.9792 | 4.36 | 6.9549 | 0.2758 | 11 | 0.9720 | 0.17 | 100% | 0.9251 | 0.27 | 100% |
| | SGS | 432 | PLS | 9 | 0.9827 | 3.98 | 0.9796 | 4.33 | 7.0069 | 0.2699 | 11 | 0.9725 | 0.17 | 100% | 0.9253 | 0.27 | 100% |
| | MFS | 432 | PLS | 9 | 0.9840 | 3.83 | 0.9806 | 4.22 | 7.1971 | 0.2963 | 11 | 0.9781 | 0.15 | 100% | 0.9346 | 0.26 | 100% |
| | GFS | 432 | PLS | 9 | 0.9846 | 3.75 | 0.9807 | 4.21 | 7.2039 | 0.2535 | 11 | 0.9809 | 0.14 | 100% | 0.9401 | 0.24 | 100% |
| | MC | 432 | PLS | 9 | 0.9856 | 3.64 | 0.9812 | 4.15 | 7.3107 | 0.2511 | 11 | 0.9825 | 0.13 | 100% | 0.9426 | 0.24 | 100% |
| | SG1D | 432 | PLS | 5 | 0.9702 | 5.22 | 0.9459 | 7.04 | 4.3744 | 1.2819 | 7 | 0.9721 | 0.17 | 100% | 0.9022 | 0.31 | 97.62% |
| | SG2D | 432 | PLS | 5 | 0.9703 | 5.22 | 0.9466 | 7.00 | 4.4085 | 1.3526 | 7 | 0.9732 | 0.16 | 100% | 0.9010 | 0.31 | 97.62% |
| | DT | 432 | PLS | 6 | 0.9671 | 5.49 | 0.9601 | 6.05 | 5.0224 | 0.4859 | 10 | 0.9826 | 0.13 | 100% | 0.9284 | 0.27 | 100% |
| | **RAW** | **432** | PLS | **8** | **0.9872** | **3.42** | **0.9832** | **3.92** | **7.8392** | **0.6914** | **10** | **0.9876** | **0.11** | **99.05%** | **0.9698** | **0.17** | **100%** |
| | ABS | 432 | PLS | 9 | 0.9847 | 3.74 | 0.9815 | 4.12 | 7.4441 | 0.6587 | 11 | 0.9870 | 0.11 | 99.05% | 0.9679 | 0.18 | 100% |
| | KM | 432 | PLS | 9 | 0.9842 | 3.80 | 0.9797 | 4.31 | 7.0326 | 0.2698 | 9 | 0.9427 | 0.24 | 98.10% | 0.9186 | 0.29 | 100% |
| | NOR | 432 | PLS | 8 | 0.9823 | 4.03 | 0.9804 | 4.24 | 7.1967 | 0.5368 | 11 | 0.9888 | 0.11 | 99.05% | 0.9684 | 0.18 | 100% |
| | MSC | 432 | PLS | 8 | 0.9859 | 3.60 | 0.9845 | 3.76 | 8.1599 | 0.6431 | 10 | 0.9883 | 0.11 | 99.05% | 0.9688 | 0.18 | 100% |
| | BC | 432 | PLS | 8 | 0.9820 | 4.06 | 0.9806 | 4.22 | 7.2681 | 0.6869 | 11 | 0.9841 | 0.13 | 99.05% | 0.9643 | 0.19 | 100% |
| HSI-NIR ($N_{T/V}$=105/105) | SNV | 432 | PLS | 8 | 0.9859 | 3.60 | 0.9845 | 3.77 | 8.1598 | 0.6417 | 10 | 0.9883 | 0.11 | 99.05% | 0.9688 | 0.18 | 100% |
| | MAS | 432 | PLS | 9 | 0.9840 | 3.83 | 0.9808 | 4.19 | 7.3590 | 0.7946 | 12 | 0.9858 | 0.12 | 99.05% | 0.9664 | 0.18 | 100% |
| | SGS | 432 | PLS | 9 | 0.9843 | 3.80 | 0.9813 | 4.14 | 7.4451 | 0.7937 | 12 | 0.9859 | 0.12 | 99.05% | 0.9665 | 0.18 | 100% |
| | MFS | 432 | PLS | 9 | 0.9855 | 3.65 | 0.9820 | 4.06 | 7.5820 | 0.7399 | 11 | 0.9815 | 0.14 | 99.05% | 0.9651 | 0.19 | 100% |
| | GFS | 432 | PLS | 9 | 0.9872 | 3.42 | 0.9832 | 3.92 | 7.8392 | 0.6914 | 11 | 0.9876 | 0.11 | 99.05% | 0.9698 | 0.17 | 100% |
| | MC | 432 | PLS | 9 | 0.9872 | 3.42 | 0.9832 | 3.92 | 7.8392 | 0.6909 | 11 | 0.9876 | 0.11 | 99.05% | 0.9698 | 0.17 | 100% |
| | SG1D | 432 | PLS | 6 | 0.9792 | 4.36 | 0.9701 | 5.24 | 5.7799 | -0.1184 | 6 | 0.9647 | 0.19 | 99.05% | 0.9284 | 0.27 | 100% |
| | SG2D | 432 | PLS | 6 | 0.9800 | 4.28 | 0.9704 | 5.21 | 5.8176 | -0.1575 | 6 | 0.9645 | 0.19 | 99.05% | 0.9291 | 0.27 | 100% |
| | DT | 432 | PLS | 8 | 0.9858 | 3.61 | 0.9836 | 3.88 | 7.8627 | 0.4886 | 10 | 0.9867 | 0.12 | 99.05% | 0.9648 | 0.19 | 100% |

**Table S2:** The MEWs selected by SRC method from SG1D spectra (originated from NIR) and raw spectra (originated from HSI), respectively.

| NIR source ($N_{T/V}$ group) | Preprocessed spectra | MEWs selection Method | Number of MEWs | Specific MEWs | Wavelength reduction |
|---|---|---|---|---|---|
| NIR ($N_{T/V}$=189/21) | SG1D | SRC | 26 | 1009.98, 1012.51, 1016.31, 1027.68, 1035.24, 1044.04, 1061.56, 1062.8, 1096.31, 1117.22, 1145.29, 1147.72, 1152.57, 1173.11, 1240.93, 1289.89, 1443.64, 1468.55, 1480.38,1497.47, 1505.97, 1532.34, 1546.97, 1578.00, 1590.29, 1612.64 | 93% |
| **NIR ($N_{T/V}$=168/42)** | **SG1D** | **SRC** | **24** | **989.63, 1009.98, 1016.31, 1044.04, 1047.8, 1052.81, 1156.21, 1173.11, 1206.62, 1240.93, 1242.10, 1281.79, 1295.66, 1335.72, 1343.66, 1407.44, 1443.64, 1445.81, 1468.55, 1480.38, 1485.73, 1513.39, 1570.80, 1608.60** | **93%** |
| NIR ($N_{T/V}$=147/63) | SG1D | SRC | 26 | 953.72, 1009.98, 1012.51, 1032.72, 1041.53, 1044.04, 1082.7, 1100.01, 1122.12, 1127.01, 1173.11, 1227.96, 1289.89, 1293.35, 1311.76, 1377.43, 1407.44, 1468.55, 1480.38, 1490.01, 1497.47, 1510.21, 1546.97, 1561.51, 1578.00, 1601.50 | 93% |
| NIR ($N_{T/V}$=126/84) | SG1D | SRC | 30 | 960.16, 1030.2, 1047.8, 1087.66, 1098.78, 1173.11,1183.92, 1206.62, 1238.58, 1279.47, 1293.35, 1295.66, 1309.46, 1345.93, 1403.01, 1420.66, 1422.86, 1443.64, 1476.08, 1480.38, 1500.66, 1534.44, 1546.97, 1549.05, 1558.40, 1566.68, 1587.23, 1589.27, 1610.62, 1648.71 | 92% |
| NIR ($N_{T/V}$=105/105) | SG1D | SRC | 22 | 965.3, 1012.51, 1015.05, 1016.31, 1153.79, 1173.11, 1192.3, 1269.01, 1295.66, 1308.32, 1361.72, 1425.05, 1443.64, 1465.32, 1466.40, 1468.55, 1546.97, 1552.17, 1601.50, 1612.64, 1624.74, 1640.75 | 94% |
| HSI-NIR ($N_{T/V}$=189/21) | RAW | SRC | 29 | 949.945, 951.591, 1009.192, 1019.064, 1032.225, 1037.16, 1060.19, 1073.349, 1094.732, 1102.955, 1142.428, 1165.453, 1168.743, 1176.966, 1257.572, 1293.774, 1313.525, 1369.509, 1379.393, 1382.687, 1392.573, 1428.83, 1437.073, 1511.314, 1577.399, 1584.013, 1608.825, 1623.719, 1660.152 | 93% |
| HSI-NIR ($N_{T/V}$=168/42) | RAW | SRC | 26 | 949.945, 994.383, 1009.192, 1019.064, 1094.732, 1099.666, 1111.179, 1121.047, 1142.428, 1163.809, 1165.453, 1178.611, 1185.190, 1234.538, 1257.572, 1293.774, 1339.866, 1369.509, 1379.393, 1382.687, 1385.982, 1395.868, 1511.314, 1584.013, 1623.719, 1660.152 | 94% |
| **HSI-NIR ($N_{T/V}$=147/63)** | **RAW** | **SRC** | **23** | **1009.192, 1020.709, 1037.16, 1073.349, 1094.732, 1102.955, 1129.27, 1145.717, 1168.743, 1198.349, 1257.572, 1313.525, 1359.627, 1362.921, 1369.509, 1379.393, 1387.63, 1392.573, 1475.006, 1557.564, 1577.399, 1593.936, 1633.652** | **95%** |
| HSI-NIR ($N_{T/V}$=126/84) | RAW | SRC | 30 | 949.945, 1004.256, 1035.515, 1102.955, 1106.245, 1158.874, 1165.453, 1168.743, 1244.409, 1257.572, 1270.735, 1277.317, 1300.357, 1326.695, 1333.28, 1343.16, 1369.509, 1372.804, 1382.687, 1387.63, 1392.573, 1521.221, 1537.737, 1567.48, 1593.936, 1597.244, 1623.719, 1633.652, 1640.275, 1660.152 | 93% |
| HSI-NIR ($N_{T/V}$=105/105) | RAW | SRC | 24 | 949.945, 1019.064, 1043.741, 1132.56, 1142.428, 1152.296, 1165.453, 1168.743, 1178.611, 1224.668, 1234.538, 1241.119, 1290.482, 1329.988, 1333.28, 1369.509, 1379.393, 1446.966, 1448.615, 1488.206, 1508.012, 1579.052, 1613.789, 1660.152 | 94% |

**Table S3:** Predicting ADA levels in WF using MEWs based on the different numbers of WF samples in training set and validation set.

| NIR source ($N_{T/V}$ group) | Preprocessed spectra | Number of MEWs | Modeling algorithm | Quantitative Regression Analysis | | | | | | | | Discriminant Analysis (whether excessive ADA) | | | | | | |
|---|---|---|---|---|---|---|---|---|---|---|---|---|---|---|---|---|---|---|
| | | | | LVs | Training set | | Validation set | | | | ΔE | LVs | Training set | | | Validation set | | |
| | | | | | $R^2_T$ | $RMSE_T$ | $R^2_V$ | $RMSE_V$ | RPD | Bias | | | $R^2_T$ | $RMSE_T$ | AOC | $R^2_V$ | $RMSE_V$ | AOC |
| NIR ($N_{T/V}$=189/21) | **SG1D** | 26 | PLS | 5 | 0.9875 | 3.38 | 0.9858 | 3.61 | 8.4333 | -0.4003 | 0.23 | 4 | 0.9328 | 0.26 | 98.41% | 0.8917 | 0.33 | 95.24% |
| | | | MLR | — | 0.9908 | 2.91 | 0.9891 | 3.16 | 9.6827 | -0.4378 | 0.25 | — | 0.9543 | 0.21 | 98.94% | 0.9209 | 0.28 | 100% |
| **NIR ($N_{T/V}$=168/42)** | **SG1D** | **24** | PLS | 4 | 0.9810 | 4.18 | 0.9819 | 4.08 | 7.4889 | -0.5363 | 0.10 | 3 | 0.9091 | 0.30 | 98.21% | 0.9119 | 0.30 | 97.62% |
| | | | **MLR** | **—** | **0.9914** | 2.81 | **0.9898** | 3.06 | **9.9001** | **-0.1769** | 0.25 | **—** | **0.9410** | **0.24** | **99.40%** | **0.9369** | **0.25** | **100%** |
| NIR ($N_{T/V}$=147/63) | **SG1D** | 26 | PLS | 5 | 0.9844 | 3.78 | 0.9607 | 6.00 | 5.1775 | 1.3526 | 2.22 | 5 | 0.9333 | 0.26 | 97.96% | 0.9094 | 0.30 | 98.41% |
| | | | MLR | — | 0.9908 | 2.90 | 0.9887 | 4.70 | 6.6141 | 1.0849 | 1.80 | — | 0.9509 | 0.22 | 99.32% | 0.9229 | 0.28 | 100% |
| NIR ($N_{T/V}$=126/84) | **SG1D** | 30 | PLS | 6 | 0.9894 | 3.12 | 0.9839 | 3.84 | 7.8833 | 0.1407 | 0.72 | 5 | 0.9253 | 0.27 | 97.62% | 0.9344 | 0.26 | 98.81% |
| | | | MLR | — | 0.9947 | 2.54 | 0.9907 | 2.92 | 10.3561 | -0.0071 | 0.39 | — | 0.9578 | 0.21 | 99.21% | 0.9464 | 0.23 | 100% |
| NIR ($N_{T/V}$=105/105) | **SG1D** | 22 | PLS | 5 | 0.9849 | 3.72 | 0.9754 | 4.74 | 6.3994 | 0.3491 | 1.02 | 3 | 0.9109 | 0.30 | 99.05% | 0.8809 | 0.34 | 98.10% |
| | | | MLR | — | 0.9884 | 3.26 | 0.9789 | 4.40 | 6.8888 | 0.1006 | 1.13 | — | 0.9458 | 0.23 | 99.05% | 0.9278 | 0.27 | 98.10% |
| HSI-NIR ($N_{T/V}$=189/21) | RAW | 29 | PLS | 9 | 0.9920 | 2.71 | 0.9854 | 3.66 | 8.3023 | 0.2916 | 0.95 | 8 | 0.8985 | 0.32 | 96.83% | 0.8792 | 0.35 | 100% |
| | | | MLR | — | 0.9944 | 2.27 | 0.9848 | 3.73 | 8.1736 | 0.4101 | 1.45 | — | 0.9203 | 0.28 | 98.94% | 0.8585 | 0.38 | 100% |
| HSI-NIR ($N_{T/V}$=168/42) | RAW | 26 | PLS | 8 | 0.9922 | 2.68 | 0.9838 | 3.86 | 8.0896 | 0.9310 | 1.18 | 8 | 0.8992 | 0.32 | 98.81% | 0.8997 | 0.32 | 100% |
| | | | MLR | — | 0.9942 | 2.31 | 0.9848 | 3.74 | 8.1754 | 0.5071 | 1.43 | — | 0.9157 | 0.29 | 100% | 0.9018 | 0.31 | 100% |
| **HSI-NIR ($N_{T/V}$=147/63)** | **RAW** | **23** | PLS | 7 | 0.9892 | 3.15 | 0.9835 | 3.88 | 7.7972 | 0.1024 | 0.74 | 8 | 0.9038 | 0.31 | 98.64% | 0.8765 | 0.35 | 100% |
| | | | **MLR** | **—** | **0.9919** | 2.72 | **0.9837** | 3.86 | **7.8388** | **0.0836** | 1.14 | **—** | **0.9101** | **0.30** | **99.32%** | **0.8787** | **0.35** | **100%** |
| HSI-NIR ($N_{T/V}$=126/84) | RAW | 30 | PLS | 9 | 0.9923 | 2.66 | 0.9801 | 4.27 | 7.1218 | 0.4427 | 1.62 | 8 | 0.8816 | 0.34 | 96.03% | 0.8517 | 0.38 | 96.43% |
| | | | MLR | — | 0.9937 | 2.40 | 0.9795 | 4.33 | 7.0018 | 0.2873 | 1.93 | — | 0.9122 | 0.30 | 98.41% | 0.8354 | 0.41 | 97.62% |
| HSI-NIR ($N_{T/V}$=105/105) | RAW | 24 | PLS | 9 | 0.9913 | 2.83 | 0.9825 | 4.00 | 7.5800 | 0.2076 | 1.17 | 7 | 0.8892 | 0.33 | 96.19% | 0.8860 | 0.34 | 97.14% |
| | | | MLR | — | 0.9944 | 2.26 | 0.9827 | 3.98 | 7.5990 | -0.01286 | 1.72 | — | 0.9201 | 0.28 | 99.05% | 0.8905 | 0.33 | 100% |
